# Evolution, expansion and expression of the Kunitz/BPTI gene family associated with long-term blood feeding in *Ixodes Scapularis*

**DOI:** 10.1186/1471-2148-12-4

**Published:** 2012-01-14

**Authors:** Shao-Xing Dai, Ai-Di Zhang, Jing-Fei Huang

**Affiliations:** 1 School of Life Sciences, University of Science and Technology of China, Hefei, Anhui 230027, P.R. China; 2 State Key Laboratory of Genetic Resources and Evolution, Kunming Institute of Zoology, Chinese Academy of Sciences, 32, Eastern Jiaochang Road, Kunming, Yunnan 650223, P.R. China

## Abstract

**Background:**

Recent studies of the tick saliva transcriptome have revealed the profound role of salivary proteins in blood feeding. Kunitz/BPTI proteins are abundant in the salivary glands of ticks and perform multiple functions in blood feeding, such as inhibiting blood coagulation, regulating host blood supply and disrupting host angiogenesis. However, Kunitz/BPTI proteins in soft and hard ticks have different functions and molecular mechanisms. How these differences emerged and whether they are associated with the evolution of long-term blood feeding in hard ticks remain unknown.

**Results:**

In this study, the evolution, expansion and expression of Kunitz/BPTI family in *Ixodes scapularis *were investigated. Single- and multi-domain Kunitz/BPTI proteins have similar gene structures. Single-domain proteins were classified into three groups (groups I, II and III) based on their cysteine patterns. Group I represents the ancestral branch of the Kunitz/BPTI family, and members of this group function as serine protease inhibitors. The group I domain was used as a module to create multi-domain proteins in hard ticks after the split between hard and soft ticks. However, groups II and III, which evolved from group I, are only present and expanded in the genus *Ixodes*. These lineage-specific expanded genes exhibit significantly higher expression during long-term blood feeding in *Ixodes scapularis*. Interestingly, functional site analysis suggested that group II proteins lost the ability to inhibit serine proteases and evolved a new function of modulating ion channels. Finally, evolutionary analyses revealed that the expansion and diversification of the Kunitz/BPTI family in the genus *Ixodes *were driven by positive selection.

**Conclusions:**

These results suggest that the differences in the Kunitz/BPTI family between soft and hard ticks may be linked to the evolution of long-term blood feeding in hard ticks. In *Ixodes*, the lineage-specific expanded genes (Group II and III) lost the ancient function of inhibiting serine proteases and evolved new functions to adapt to long-term blood feeding. Therefore, these genes may play a profound role in the long-term blood feeding of hard ticks. Based our analysis, we propose that the six genes identified in our study may be candidate target genes for tick control.

## Background

Ticks are classified into two major families: Ixodidae (hard ticks) and Argasidae (soft ticks) [1,2]. The family Ixodidae is further divided into two groups, Prostriata and Metastriata. Prostriata contains only a single genus, *Ixodes*. In contrast, Metastriata contains four subfamilies: Amblyomminae, Haemaphysalinae, Hyalomminae, and Rhipicephalinae [1,2]. All ticks are external blood-feeding parasites of mammals, birds and reptiles throughout the world [3,4]. They can transmit a wide variety of pathogens causing several human and animal diseases, including Lyme disease, human granulocytic anaplasmosis, and human babesiosis [5,6]. However, hard and soft ticks display different feeding strategies. Hard ticks feed on blood for a few days to over one week, whereas soft ticks typically feed on blood for minutes to hours [7]. The evolutionary drivers of long-term blood feeding in hard ticks remain unknown.

Blood feeding is a complex process. When attempting to feed the blood from their hosts, ticks face the problem of host defenses, such as hemostasis, inflammation, and immunity [7-10]. Recent studies of the saliva transcriptome of ticks [11-20] and some review papers [7,10,21] have demonstrated that tick salivary proteins play a profound role in the process of blood feeding. Kunitz/BPTI proteins are abundant in the salivary glands (SGs) of ticks [11-18], suggesting that they have important roles in blood feeding. The Kunitz/BPTI domain is an ancient and widespread domain with a disulfide-rich alpha + beta fold that is stabilized by three highly conserved disulfide bridges with the bonding patterns 1-6, 2-4, and 3-5 [22-24]. The typical Kunitz/BPTI domain has a cysteine pattern of CX(8)CX(15)CX(7)CX(12)CX(3)C [22-24]. Ticks exhibit other cysteine patterns, such as CX(8)CX(18)CX(5)CX(12)CX(3)C and CX(5,6)CX(15)CX(8)CX(11)CX(3)C, in the Kunitz/BPTI proteins due to insertions and deletions (indels) [12,15]. Additionally, Kunitz/BPTI proteins in the SGs and midgut of ticks have signal peptides that allow them to be secreted into the extracellular medium [15,25]. Interestingly, the Kunitz/BPTI domain was used as a module to construct multi-domain Kunitz/BPTI proteins in ticks. Therefore, some tick proteins have complex domain architectures containing two or more Kunitz/BPTI domains [12,15]. The domain architectures and sequences of the Kunitz/BPTI proteins are highly divergent between soft and hard ticks [8,12,15]. Furthermore, the various Kunitz/BPTI proteins can perform different functions. In soft ticks, Kunitz/BPTI proteins function as anti-hemostatic factors by inhibiting blood coagulation and platelet aggregation [7,8,26]. In hard ticks, Kunitz/BPTI proteins can regulate host blood supply [24] and disrupt host angiogenesis and wound healing [27].

How the functional differences and complex domain architectures of Kunitz/BPTI proteins emerged and whether this evolution is associated with differences in blood-feeding strategies between soft and hard ticks remain unclear. Studying the evolution and expression of Kunitz/BPTI gene family between soft and hard ticks can help answer these questions. The evolution of the Kunitz/BPTI family in soft ticks has been previously studied [11,26]. These studies revealed that the blood coagulation and platelet aggregation inhibitors (Kunitz/BPTI proteins) evolved in the ancestral soft tick lineage and persisted in all soft ticks. However, to our knowledge, there has not been a comprehensive and systematic study on the evolution and expression of Kunitz/BPTI gene family in hard ticks. The hard tick *Ixodes scapularis *and its close relatives, *Ixodes pacificus *and *Ixodes ricinus*, are the most important ticks because they transmit the majority of emerging human disease pathogens [28,29]. The genome and saliva transcriptome of *Ixodes scapularis *are now available [15,18,28], which provides an opportunity to study the evolution and expression of Kunitz/BPTI gene family in *Ixodes scapularis*.

In our study, we systematically examined the evolution, expansion and expression of Kunitz/BPTI gene family in *Ixodes scapularis*. We then compared the characteristics of this family between soft and hard ticks by sequence, structure and gene expression analyses. Based on these analyses, we illustrated the profound role of this family in long-term blood feeding and discuss the reasons for different blood-feeding strategies between hard and soft ticks. Finally, we proposed that the six genes (Table 1) with highly dynamic expression identified in our study might be candidate target genes for tick control.

**Table 1:** Six differentially expressed Kunitz/BPTI genes in *Ixodes scapularis*.

Unigene	Protein	R	ISUF	IS6-12	IS18-24	ISA72
**Isc.218**	AAY66618.1 (group II)	241.40	0	17	259	2

**Isc.190**	AAM93606.1(group II)	19.74	0	0	0	18

**Isc.255**	AAM93612.1(group II)	10.96	0	0	0	10

**Isc.196**	AAM93610.1(group II)	10.96	0	0	0	10

**Isc.180**	AAM93635.1(group III)	23.03	0	0	0	10

**Isc.179**	AAM93632.1(group III)	9.87	0	0	0	9

Note. This table lists the 6 genes with R > 9. The first column is the name of Unigene cluster. The second column is protein corresponding to Unigene cluster, with the classification in bracket. The third column gives the likelihood statistic R. The next four columns show the number of ESTs in each of the four libraries that belong to the Unigene cluster. The total numbers of EST sequences in each of the libraries are 1026, 952, 1995 and 1993.

## Results

### Identification, classification and gene structure of the Kunitz/BPTI family in all ticks

The sequence search strategy used here was described in Methods and additional file (Additional File 1). We retrieved 368 sequences in the NR database at the NCBI website. After removing redundant sequences (Additional File 2), partial sequences and other sequences without the cysteine motif of the Kunitz/BPTI domain, a total of 303 Kunitz/BPTI proteins were recognized in ticks and used for analysis in this study. Among the 303 sequences, there are 162 single-domain Kunitz/BPTI proteins (One-KU), 92 two-domain Kunitz/BPTI proteins (Two-KU), 16 three-domain Kunitz/BPTI proteins (Three-KU), 9 four-domain Kunitz/BPTI proteins (Four-KU), 17 five-domain Kunitz/BPTI proteins (Five-KU), and 3 seven-domain Kunitz/BPTI proteins (Seven-KU). Six-KU proteins were not present. Signal peptides of the 303 sequences were predicted using the SignalP 4.0 server [30]. We found that most of the Kunitz/BPTI proteins (205/303 sequences) have signal peptides. Signal peptides were detected in each type of Kunitz/BPTI protein, indicating that Kunitz/BPTI proteins perform their function mainly in the extracellular medium.

**Figure 1:**
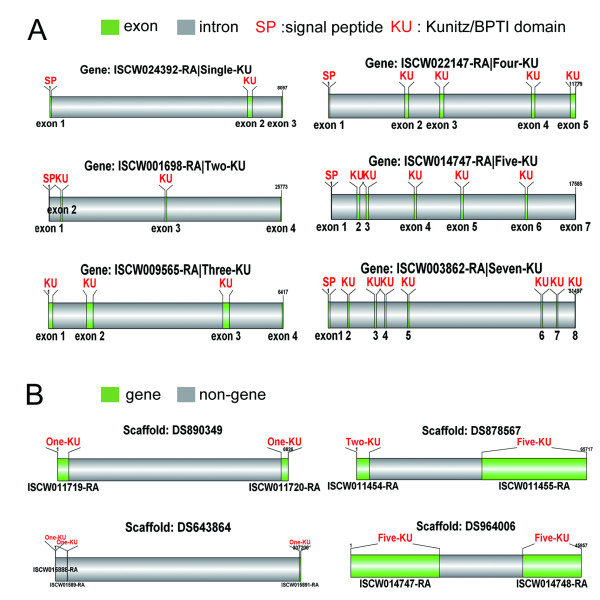
**Gene structures of Kunitz/BPTI proteins and their distributions in the genome**. **(A)**, Gene structures of Kunitz/BPTI proteins. The gene structures of single and multi-domain Kunitz/BPTI proteins show similar characters. Exons were colored with green and numbered below. SP: signal peptide; KU: Kunitz/BPTI domain; **(B)**, the distributions of Kunitz/BPTI gene. Some Kunitz/BPTI genes are arranged in tandem in the genome. Genes were colored with green.

The genome data of *Ixodes scapularis *contain rich information about the organization between Kunitz/BPTI gene loci and their gene structures. By aligning mRNA to genomic sequences, we obtained gene structures and organization for the Kunitz/BPTI gene loci (Figure 1). Single-domain proteins have similar gene structure. Furthermore, single and multi-domain Kunitz/BPTI proteins have similar gene structure. The first exon encodes a signal peptide, and the Kunitz/BPTI domain is encoded in a single exon (Figure 1A). Multiple exons arrayed in tandem in the gene contribute to multi-domain Kunitz/BPTI proteins. The length of the introns is divergent. However, the Kunitz/BPTI domain-encoding exon has an average length of 180 bp, which encodes the 60-aa domain. Some Kunitz/BPTI genes (9/55) are arranged in tandem in the genome (Figure 1B), while other Kunitz/BPTI genes (46/55) are dispersed throughout the genome.

Additionally, we obtained functional information for the Kunitz/BPTI proteins [20,25,31-34]. All 303 sequences are listed with related information, such as the species name, gene name, domain architecture, length, signal peptide and function (Additional File 3).

### Evolution of single-domain Kunitz/BPTI proteins: Evidence for lineage-specific expansion in *Ixodes *ticks

**Figure 2:**
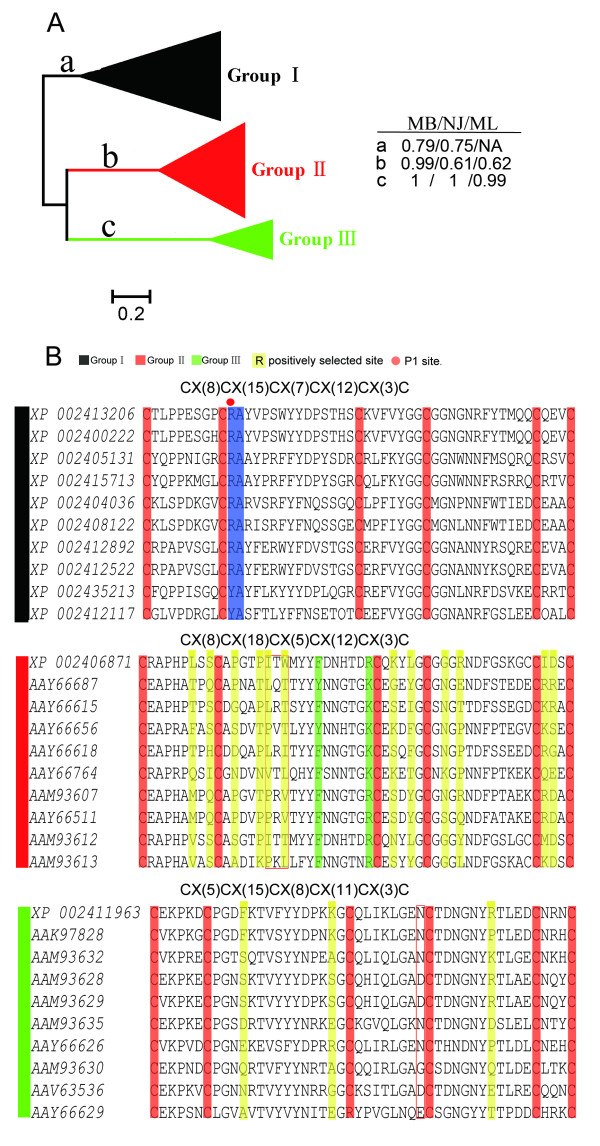
**Cluster and alignment of single-domain Kunitz/BPTI proteins in *Ixodes scapularis***. **(A)**, MB tree for single-domain Kunitz/BPTI proteins in *Ixodes scapularis*. The length of each branch is proportional to the estimated number of substitutions. Bar denotes 20% substitutions per site. The support values for each group (in MB, NJ, and ML tree) were shown in right panel. "NA" mans that proteins in group1 do not cluster into one clade (the bootstrap values < 50%) in the ML tree of single-domain Kunitz/BPTI proteins in *Ixodes scapularis*. (Note: the value 50% is cutoff value in the Tree Finder.) (B), Alignment of representing single-domain Kunitz/BPTI proteins in *Ixodes scapularis*. The alignment only shows the part between the first and sixth cysteine (C1-C6) in Kunitz/BPTI protein. Red boxes indicate different insertions relative to group I. The six conserved cysteines were colored by red. Red circle indicates the position of P_1 _site. Key residues for serine proteases inhibition and ion channels modulating were highlighted in blue and green, respectively. Positively selected sites in group II and III were colored by yellow.

To clarify the phylogenetic relationships of single-domain Kunitz/BPTI proteins in *Ixodes scapularis *and compare them with other tick Kunitz/BPTI proteins, we first analyzed the Kunitz/BPTI proteins in *Ixodes scapularis*. Then, all tick Kunitz/BPTI proteins were gathered for further analysis. A total of 80 Kunitz/BPTI proteins in *Ixodes scapularis *are clustered into three clades (named groups I, II and III) (Figure 2A and Additional File 4). Groups II and III are highly supported with posterior probabilities of 0.99 and 1 in Bayesian inference (MB) tree and bootstrap values of 61% and 100% (NJ tree) and 62% and 100% (ML tree), respectively. In contrast, group I forms one clade with lower support values of 0.79 in the MB tree and 0.75 in the NJ tree. The proteins group I do not cluster into one clade in the ML tree (Additional File 4). The Low supports for the three groups may be due to the long branches and divergence in the group. Groups I, II and III follow the cysteine patterns CX(8)CX(15)CX(7)CX(12)CX(3)C, CX(8)CX(18)CX(5)CX(12)CX(3)C and CX(5,6) CX(15)CX(8)CX(11)CX(3)C, respectively, although there are exceptions(Figure 2B and Additional File 5). The pattern of group I is the typical cysteine pattern of the Kunitz/BPTI family, and this motif is also common in Bilateria and Cnidaria [24,35-39]. This suggests that group I represents the ancestral branch of the Kunitz/BPTI family in *Ixodes scapularis*.

**Figure 3:**
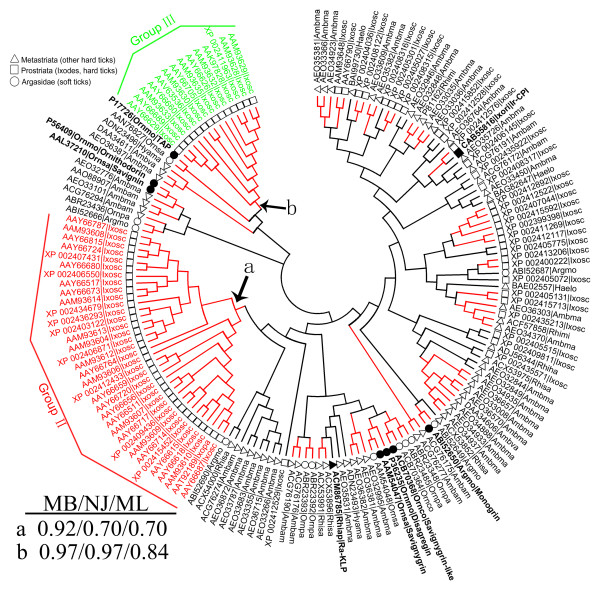
**Neighbor-joining tree for all single-domain Kunitz/BPTI proteins in ticks**. The circular phylogram (NJ tree) is based on alignment of all single-domain Kunitz/BPTI proteins in *Ixodes scapularis *and other ticks. The red branches have bootstrap support above 50%. Group II and group III, which are highlighted, are lineage-specific expansions of Kunitz/BPTI family in the genus *Ixodes*. The support values for these two groups (in MB, NJ, and ML tree) were shown in left bottom. The circle, square and triangle in the phylogram indicate the proteins from soft ticks, the genus *Ixodes *(hard ticks) and other hard ticks, respectively. The sequence name consists of 5 letters (3 from the genus and 2 from the species name) with the NCBI accession number. (Argmo, *Argas monolakensis*; Ornco, *Ornithodoros coriaceus*; Ornmo, *Ornithodoros moubata*; Ornpa, *Ornithodoros parkeri*; Ornsa, *Ornithodoros savignyi*; Rhiap, *Rhipicephalus appendiculatus*; Rhisa, *Rhipicephalus sanguineus*; Ixosc, *Ixodes scapularis*; Ixopa, *Ixodes pacificus*; Ixori, *Ixodes ricinus*; Rhimi, *Rhipicephalus microplus*; Hyama, *Hyalomma marginatum rufipes*; Ambam, *Amblyomma americanum*; Haelo, *Haemaphysalis longicornis*).

Next, we extended our analysis of single-domain Kunitz/BPTI proteins from *Ixodes scapularis *to all ticks. Group I proteins in *Ixodes scapularis *are distributed widely, and they do not form a monophyletic clade (Figure 3 and Additional File 6), which further indicates that group I represents the ancestral branch of the Kunitz/BPTI family in *Ixodes scapularis*. By contrast, groups II and III form two separate monophyletic clades with high support (posterior probabilities of 0.92 and 0.97 in the MB tree and bootstrap values of 70% and 97% in the NJ tree and 70% and 84% in the ML tree, respectively) (Figure 3 and Additional File 6). This suggests that group II and III proteins may be only present in the genus *Ixodes*.

To confirm this argument, we searched the EST database of NCBI by TBLASTN. We were able to obtain some Kunitz/BPTI ESTs in *Ixodes ricinus *with translated protein sequences containing cysteine patterns from groups II and III (Additional File 7). However, this cysteine pattern was not found in other tick genera ESTs. Furthermore, we searched the genome of an additional hard tick (*Rhipicephalus microplus*) using TBLASTN but did not find any group II or III proteins. Taken together, we infer that the genes in groups II and III are only present in species of the genus *Ixodes*. Groups II and III are lineage-specific expansions of the Kunitz/BPTI family in *Ixodes*.

### Evolution of multi-domain Kunitz/BPTI proteins: The group I domain is a module for constructing multi-domain Kunitz/BPTI proteins

**Figure 4:**
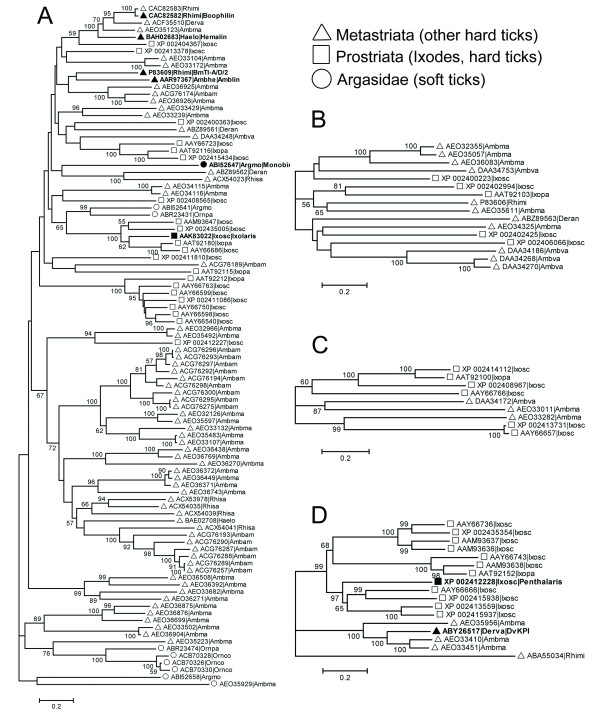
**Neighbor-joining trees for multi-domain Kunitz/BPTI proteins in ticks**. Neighbor-joining trees for Two-KU **(A)**, Three-KU **(B)**, Four-KU **(C) **and Five-KU **(D) **proteins in ticks were reconstructed. The length of each branch is proportional to the estimated number of substitutions. Bar denotes 20% substitutions per site. The bootstrap values (> 50%) were shown in branches. The circle, square and triangle in the phylogram indicate the proteins from soft ticks, the genus *Ixodes *(hard ticks) and other hard ticks, respectively. The sequence name consists of 5 letters (3 from the genus and 2 from the species name) with the NCBI accession number.

There are many multi-domain Kunitz/BPTI proteins in ticks. Two-, three-, four- and five-KU proteins from all available tick sequences were aligned, and their corresponding phylogenetic trees were constructed. A phylogenetic tree for seven-KU proteins was not constructed because only three seven-KU proteins were found (one in *Ixodes scapularis *and two in *Amblyomma maculatum*). By carefully checking these phylogenetic trees, we found that two-KU proteins are widespread in both soft and hard ticks (Figure 4A). Intriguingly, the proteins that have more than two Kunitz/BPTI domains (three-, four-, five- and seven-KU) are only present in hard ticks (Figure 4B, C, and 4D). This indicates that many multi-domain Kunitz/BPTI proteins were created in hard ticks during the evolution of the Kunitz/BPTI family. These multi-domain Kunitz/BPTI proteins may contribute to the different blood-feeding strategies of soft and hard ticks.

In our analysis, multiple Kunitz/BPTI domains in tandem contribute to multi-domain Kunitz/BPTI proteins. Because single-domain Kunitz/BPTI proteins were classified into three groups, we examined whether multi-domain Kunitz/BPTI proteins arose from a single Kunitz/BPTI group or whether they originated from different Kunitz/BPTI groups. First, all multi-domain Kunitz/BPTI proteins in *Ixodes scapularis *were split into single-domain segments. Then, these single-domain segments were aligned together with all of the single-domain Kunitz/BPTI proteins in *Ixodes scapularis*, and the corresponding phylogenetic trees were constructed (Additional File 8). Again, groups II and III form two separate monophyletic clades with high support (posterior probabilities of 0.96 and 1 in the MB tree and bootstrap values of 77% and 96% in the NJ tree and 82% and 91% in the ML tree, respectively). In contrast, group I proteins are distributed widely, and they cluster with other domains from the different multi-domain Kunitz/BPTI proteins. This indicates that only the group I domain was used as a module for constructing multi-domain Kunitz/BPTI proteins, and group II and III proteins were not involved in the formation of multi-domain Kunitz/BPTI proteins.

This hypothesis is also supported by the species distribution of the Kunitz/BPTI proteins. Groups II and III are only present in the genus *Ixodes*. However, multi-domain Kunitz/BPTI proteins are widely present in hard ticks (including the genera *Ixodes*, *Amblyomma*, and *Rhipicephalus*) and soft ticks (in the case of two-KU proteins). This suggests that multi-domain Kunitz/BPTI proteins emerged earlier than the development of group II and III proteins. Therefore, it is impossible for multi-domain Kunitz/BPTI proteins to have originated from group II and III domains.

### Functional site analysis of the Kunitz/BPTI family in *Ixodes scapularis*

**Figure 5:**
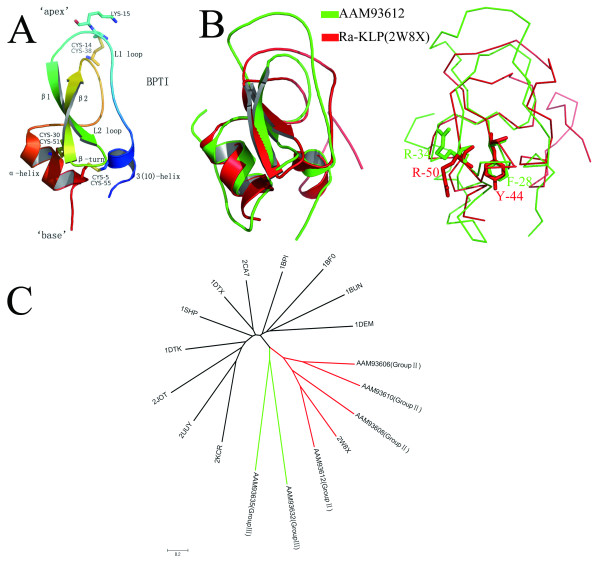
**Structure of classical Kunitz/BPTI protein and structural alignment of group II protein and Ra-KLP**. **(A)**, Structure of classical Kunitz/BPTI domain (PDB code 1BPI). Secondary structural elements were colored from blue (N-terminus) to red (C-terminus). The three conserved disulphide bridges and the P_1 _residue (Lys15 in BPTI) are shown as an atom-colored stick model. **(B)**, Structural alignment of proteins AAM93612 (group II) and Ra-KLP (PDBID: 2W8X). The two proteins share strong similarities in the regions of 3^10 ^helix and β-turn at the "base" of the structure. In contrast, there is significant difference at the "apex" of the structure between the two proteins, due to different type indels. The superimposed structures are shown as cartoon (left) and ribbon (right). The key residues associated with channel-modulating activity are represented as stick (AAM93612: R-34, F-28; Ra-KLP: R-50, Y-44). **(C)**, Structure-based neighbor-joining (NJ) tree of Kunitz/BPTI proteins. Six protein modes (AAM93606, AAM93608, AAM93610 and AAM93612 from group II; AAM93632 and AAM93635 from group III) were aligned with other Kunitz/BPTI protein structures (1BF0, 1BPI, 1BUN, 1DEM, 1DTK, 1DTX, 1SHP, 2CA7, 2JOT, 2KCR, 2UUY, 2W8X) using the MISTRAL online server. The root-mean-square deviation (RMSD) of pairwise structures was used to reconstruct neighbor-joining (NJ) tree. The length of each branch is proportional to the RMSD. Bar denotes 20% RMSD.

The typical Kunitz/BPTI protein has a conserved tertiary structure stabilized by three highly conserved disulfide bridges with the bonding patterns 1-6, 2-4, and 3-5 (Figure 5A). The structure-function relationship of Kunitz/BPTI proteins has been studied in prior studies [22,24,34,36,37,40-43]. Kunitz/BPTI proteins have two functions. One commonly accepted function is the inhibition of serine proteases [8,44]. The second function, which is relatively rare, is the blocking of ion channels [24,45]. At the "apex" of the inhibitor structure, a protruding loop (L1) penetrates deeply in the catalytic-site cleft of a protease to inhibit the protease via the P_1 _residue side chain (Lys15 in BPTI) [24,36,46,47] (Figure 5A). In ion channel blockers, the "base" of the structure, which contains a 3^10 ^helix and a β-turn, binds near the entrance of the pore of an ion channel to block the ion channel activity [39,48-50] (Figure 5A).

The key residues for inhibiting serine proteases are the amino acids surrounding the P_1 _residue that are in direct contact with the protease [51]. Trypsin inhibitors have a basic residue (R or K) in the P_1 _position followed by a small residue (motif: C_2 _[K/R] s). Chymotrypsin inhibitors have a residue with a large side-chain (F, L, N, Y) in the P_1 _position followed by a small residue (motif: C_2 _[B] s) (where C_2_, B and s indicate the second residue cysteine, a residue with a large side-chain and a small residue, respectively) [36]. Most of the proteins in group I exhibit the motifs C_2 _[K/R] s and C_2 _[B] s (Figure 2B and Additional File 5), which suggests that they are serine protease inhibitors. Therefore, group I proteins may function as anti-hemostatic factors by inhibiting serine proteases in the coagulation system. For example, the protein Ir-CPI (Figure 3) from *Ixodes ricinus *has the motif C2 [K/R] s, and it can inhibit FXIIa, FXIa, and kallikrein [52].

However, proteins in groups II and III lack the key residues for inhibiting serine proteases (Figure 2B), suggesting that they lost this function. To investigate whether group II and III proteins have evolved new functions, we performed structure-based phylogenetic analysis (Figure 5B, C). Group III proteins form a monophyletic clade that does not include homologous structures (Figure 5C), preventing us from inferring their function. Interestingly, group II proteins and Ra-KLP (PDBID: 2W8X) [24] are clustered into the same clade (Figure 5C). Ra-KLP, which is a salivary protein in *Rhipicephalus appendiculatus *(hard tick), can regulate host blood flow and innate immunity through modulating maxiK channels [24]. Ra-KLP is well superimposed with protein AAM93612 (group II) (RMSD: 1.2) (Figure 5B). The two proteins share strong similarities in the regions of the 3^10 ^helix and the β-turn at the "base" of the structure (Figure 5B). Furthermore, structural elements (R or K and a close hydrophobic residue [L, Y, or F]) associated with channel-modulating activity [39,48] are present in the AAM93612 protein and most proteins in group II (Figure 5B and Additional File 5). These observations indicate that proteins in group II may modulate ion channels, such as maxiK channels. Some of the proteins in group II possess a Q instead of an R or K (Additional File 5), which means that these proteins cannot exhibit channel-modulating activity. Therefore, the possibility that group II may have multiple functions should not be excluded.

### The expression patterns of genes in groups II and III exhibit stage-specificity during long-term blood feeding in *Ixodes *ticks

**Figure 6:**
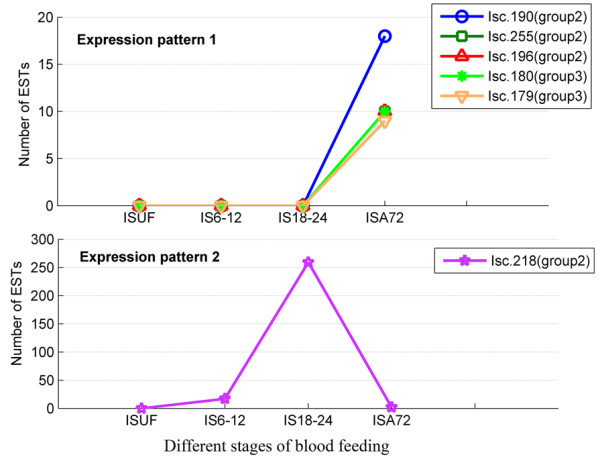
**Expression patterns of six Kunitz/BPTI genes in *Ixodes scapularis *identified in our study**. The number of ESTs for each gene is plotted against different stages of tick blood feeding (ISUF, IS6-12, S18-24 and ISA72). The details of the four stages are described in the Methods. Different genes are indicated by different colored makers. Expression of genes (Isc.190, Isc.255, Isc.196, Isc.180 and Isc.179) arises at 72 hours post host attachment (**Expression pattern 1**). However, expression of gene Isc.218 begins at 6-12 hours (17 ESTs), elevates strikingly at 18-24 hours (259 ESTs) and decreases rapidly at 72 hours (2 ESTs) post host attachment (**Expression pattern 2**).

Hard ticks feed on blood for a few days to over one week, whereas soft ticks typically feed on blood for only minutes to hours [7]. Therefore, if genes exhibit significantly higher expression at 6-12 hours or later after host attachment, such genes may have a potentially important role in the long-term blood feeding of hard ticks. Here, we used R statistics [53] to analyze four cDNA libraries to identify six differentially expressed genes (R > = 9, Believability > = 99%) belonging to the Kunitz/BPTI family during different stages of blood feeding in *Ixodes scapularis *(Table 1). All six genes were highly expressed at 6-12 hours or later after host attachment and two types of expression patterns were observed (Figure 6). The expression of genes Isc.190, Isc.255, Isc.196, Isc.180 and Isc.179 increases at 72 hours after host attachment (Figure 6**Expression pattern 1**). However, the expression of gene Isc.218 begins at 6-12 hours (17 ESTs), elevates strikingly at 18-24 hours (259 ESTs) and decreases rapidly at 72 hours (2 ESTs) after host attachment (Figure 6**Expression pattern 2**).

We further searched the EST database of *Ixodes ricinus *and found similar expression patterns for group II and III genes (Additional File 7). In both *Ixodes scapularis *and *Ixodes ricinus*, group II genes were expressed in the middle and late stages of long-term blood feeding, whereas group III genes were only expressed in the late stage of long-term blood feeding (Figure 6 and Additional File 7). The expression patterns of genes in groups II and III exhibit stage-specificity during long-term blood feeding. This suggests that these genes are functionally linked to long-term blood feeding in the *Ixodes *ticks.

### Positive selection drove the evolution of group II and III genes

**Figure 7:**
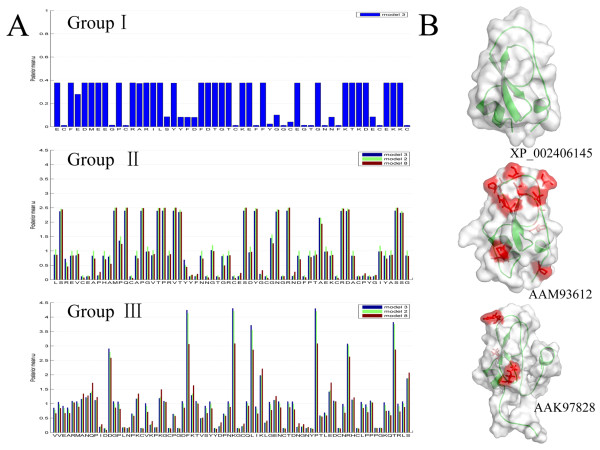
**Positive selection detection result of Kunitz/BPTI family in *Ixodes scapularis***. **(A)**, Column charts of the posterior mean ω for each site in group I, group II and group III. In these charts, the blue, green and red columns are based on M3, M2a and M8, respectively. Positively selected sites are detected in both group II and group III, but not in group I. **(B)**, Surface models of representative proteins in group I, group II and group III. Protein XP_002406145.1, AAM93612.1 and AAK97828.1 from group I, group II and group III, respectively, were modeled using the I-TASSER server. Positively selected sites with posterior probability > 95% (Table S4-S5) are located in the surface models (shown as stick with red color).

Positive selection is a major driving force in the expansion of gene families for particular functions [54]. To investigate whether positive selection drove the evolution of new functions in the Kunitz/BPTI family in *Ixodes scapularis*, site-specific models implemented in the CODEML program of PAML version 4.4c [55] were used to evaluate the selective pressure in each group. We found strong evidence (with p-value < 0.0001 for M1a vs. M2a and M7 vs. M8) for positive selection imposed on both groups II and III, and we identified positively selected sites in each group (Figure 2B, 7 and Additional File 9 and 10). However, there no evidence for positive selection was found in group I (Figure 7 and Additional File 11).

Substitution saturation could bias the estimation of the dN/dS ratios. In order to assess substitution saturation of three groups (group I, II and III) further, two methods (Steel's method [56] and Xia's method [57]) implemented in the software DAMBE [58] were used. Both methods show that group I has experienced substitution saturation, but groups II and III have experienced little substitution saturation (Additional file 12). Furthermore, previous research shows that saturation does not increase the rate of false positive predictions for the likelihood ratio test [59]. Therefore, the results of detection of positive selection within group II and III sequences remain relevant. Although Bayesian prediction of sites under positive selection is more sensitive to saturation level, the divergence levels estimated here do not pose a serious concern [60,61]. Therefore, we used detected sites under positive selection for further exploratory analyses.

Sequences used in the PAML analysis and the phylogenies with dN and dS on each branch have been provided in additional files (Additional File 13 and 14). There are more positively selected sites in group II than in group III (Figure 7, Additional File 9 and 10). To address whether the identified sites under positive selection relate to the molecular function of the Kunitz/BPTI family, the positively selected sites were mapped to three-dimensional structures modeled by I-TASSER online [62]. In group II, the positively selected sites are in the L1 and L2 loops at the "apex" and the α-helix at the "base" of the structure (Figure 7B), which suggests that there was selection for rapid changes in this region. The rapid amino acid substitutions could lead to structural conformational changes in the 3^10 ^helix and β-turn, which paves the way for the evolution of new functions, such as modulating ion channels. In group III, only three positively selected sites were identified; they are located at the L1 loop, L2 loop and β-turn (Figure 7B), which suggests that these sites are involved in the function of group III proteins. In both group II and group III, positively selected sites are located in the functional regions. Therefore, rapid evolution in these two groups of Kunitz/BPTI proteins may have contributed to the evolution of new functions.

## Discussion

### The evolution scenario of Kunitz/BPTI family in ticks

**Figure 8:**
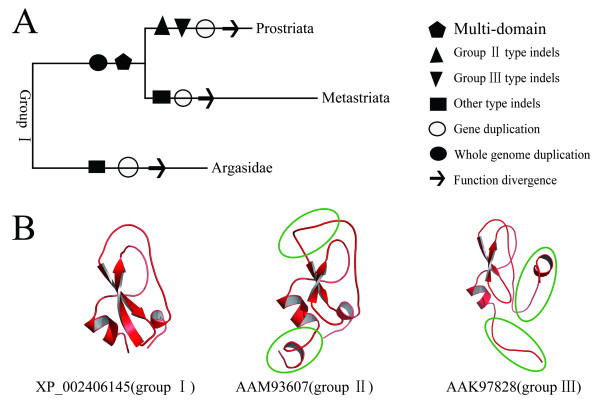
**The evolution scenario and structural variations of Kunitz/BPTI family in the ticks**. **(A)**, the evolution scenario of Kunitz/BPTI family in the ticks. Events of different indels (group II type, group III type and other type indels), gene duplication, whole genome duplication, multi-domain and function divergence, occurred during the evolution of Kunitz/BPTI family in the ticks. These events are indicated by different shapes. **(B)**, Structural variations of Kunitz/BPTI family in the ticks. The group II and group III type indels lead to structural changes of Kunitz/BPTI proteins. These changes in structure (group II, group III) relative to classical Kunitz/BPTI proteins (group I) are circled.

Ticks are classified into several groups (Figure 8). Previous studies indicated that Ixodidae (hard ticks) evolved from bird-feeding soft ticks and whole genome duplication occurred once early in tick evolution [15,63,64]. Based on our results combined with those of previous studies, the evolution of the Kunitz/BPTI family in ticks is proposed as follows (Figure 8A). The common ancestor of ticks carried Kunitz/BPTI genes orthologous to those in group I, because group I genes are present in both soft and hard ticks. Hard and soft ticks diverged between 120 and 92 MYA [26], and whole genome duplication occurred in this divergence. Many multi-domain Kunitz/BPTI proteins were created in hard ticks after the split between hard and soft ticks. Three-, four-, five- and seven-KU proteins are only present in hard ticks. After the split of Prostriata and Metastriata, certain indels developed in group I genes followed by multiple gene duplications gave rise to groups II and III in Prostriata. By contrast, other types of indels developed in group I genes in Metastriata. The group II and III type indels lead to structural variations in the Kunitz/BPTI proteins (Figure 8B). For example, the L1 loop in group II turns into a longer and distorted loop, and the 3^10 ^helix is not present in group III. These variations may result in evolutionary novelty by changing the structure and therefore the function of the protein. Then, positive selection drove the divergence of genes in groups II and III in Prostriata, as described above. And structural alignment and functional site analysis suggests that genes in group II evolved a new function of modulating ion channels.

Ra-KLP, which is a salivary protein in *Rhipicephalus appendiculatus *(Metastriata), lost the function of inhibiting serine proteases but gained the function of modulating maxiK channels [24]. Both Ra-KLP and group II proteins are members of the Kunitz/BPTI family, but they resulted from different types of indels and formed different cysteine patterns after the split between Prostriata and Metastriata (Figure 5B, 8A and Additional File 15). However, the two proteins share strong similarities in their 3^10 ^helices and β-turns at the "base" of the structures, and both have structural elements associated with channel-modulating activity (Figure 5B and Additional File 15). This is an example of the phenomenon of convergent evolution. Kunitz/BPTI proteins in the two branches of hard ticks (Prostriata and Metastriata) acquired similar structures and functions despite having different indels and cysteine patterns (Figure 5B and Additional File 15).

This evolutionary scenario is rare in other species, such as snakes, spiders, cone snails and sea anemones, in which positive selection was detected in the evolution of the Kunitz/BPTI family despite the absence of indels [24,35-39].

### The profound role of Kunitz/BPTI family in long-term blood feeding

To successfully feed to repletion, ticks must circumvent host hemostatic, inflammatory and immune responses [7-9]. Hemostatic responses rely on the triad of blood coagulation, platelet aggregation, and vasoconstriction [7]. In our study, group I proteins are serine protease inhibitors that function as anti-hemostatic factors by inhibiting kallikrein and the coagulation factors FXIIa, FXIa and FXa (Figure 3). Group II proteins may have evolved a new function in regulating host blood flow and innate immunity through modulating maxiK channels. Hard ticks imbibe large volumes of blood (approximately two thirds of their body volume) and expand enormously in the late stages of long-term blood feeding (named the rapid engorgement stage) [65,66]. Regulating host blood flow can enhance blood-feeding efficiency in the late stages of long-term blood feeding, which is beneficial for the rapid engorgement of hard ticks. Therefore, this function may play a profound role in the long-term blood feeding of hard ticks. Gene expression analysis reveals that the expression of genes in groups II and III is significantly higher in the late stages of long-term blood, which further suggests that these genes are functionally linked to long-term blood feeding.

### Different blood-feeding strategies may result from different sizes of the gene family in soft and hard ticks

Hard and soft ticks display different blood-feeding strategies: soft ticks typically feed for less than one hour, whereas hard ticks feed for prolonged periods of time, varying from a few days to over one week [7]. The reasons for this difference are not fully clarified [1,7,12]. Previous studies indicated that both hard and soft ticks possess a similar number of gene families, but they have different numbers of gene family members [12]. Multiple gene duplications occurred in hard ticks after whole genome duplication, resulting in a large number of gene family members. Compared with hard ticks, soft ticks possess fewer members in each family [12]. Therefore, different blood-feeding strategies may result from different sizes of the gene family in soft and hard ticks. In our study, there are a large number of Kunitz/BPTI genes, especially in the lineage-specific expanded genes (group II and III) in hard ticks. Group II and III proteins are functionally linked to long-term blood feeding. Furthermore, multi-domain proteins (three-, four-, five- and seven-KU) are only present in hard ticks. In contrast, the few members of the Kunitz/BPTI family and the absence of groups II and III and multi-domain proteins in soft ticks may partly explain why soft ticks cannot feed on blood for longer periods.

### The six genes with highly dynamic expression identified in our study may be candidate target genes for tick control

Tick control is important because ticks are vectors of a variety of bacterial and protozoan diseases, and ticks have the ability to transmit pathogens to vertebrates [5,6]. The development of vaccines directed against tick proteins may reduce tick infestations and the transmission of tick-borne pathogens. However, the limiting step in tick vaccine development is the identification of tick protective antigens [67-69]. Here, we propose that proteins encoded by the six genes (Table 1) identified in our study may be candidate protective antigens. This proposal is based on the following findings. First, the expression of these six genes increases significantly at 6-12 hours or later after host attachment. Additionally, these genes belong to groups II and III, which are subject to positive selection. The patterns of expression and evolution strongly suggest an important role for these genes in the process of long-term blood feeding. Second, safety issues may arise when protein ortholog sequences are used for vaccination because they can potentially induce autoimmune responses that are damaging to the host [68,70]. Fortunately, these group II and III genes are lineage specific and only present in *Ixodes *ticks. Although this feature makes these genes safe for vaccine development, it limits the application to only *Ixodes *ticks. Third, the Salp10 (AAK97828) protein in group III had been previously identified as a salivary gland antigen that elicited antibodies in the host [71]. This provides direct experimental evidence to support our proposal. Taken together, these six genes may be promising target genes for tick control. Additional experimental studies are needed to assess the validity of these genes for vaccine development.

## Conclusions

Our study reveals the great differences between soft and hard ticks in the Kunitz/BPTI family. Compared with hard ticks, soft ticks do not possess group II and III proteins and multi-domain proteins (three-, four-, five- and seven-KU). Many multi-domain Kunitz/BPTI proteins were created in hard ticks using the group I domain as a module after the split between hard and soft ticks. Groups II and III, which exhibit significantly higher expression during long-term blood feeding, are only present and expanded in the genus *Ixodes*. In *Ixodes*, positive selection drove the expansion of the Kunitz/BPTI family and the evolution of new functions in group II such as ion channel-modulating ability. The two groups may play a profound role in the long-term blood feeding of hard ticks by enhancing blood-feeding efficiency in the late stages of long-term blood feeding, which is beneficial for the rapid engorgement of hard ticks. Therefore, our results suggest that the differences in the Kunitz/BPTI family between soft and hard ticks may be linked to the evolution of long-term blood feeding in hard ticks. Finally, we propose that the six genes (Isc.218, Isc.190, Isc.255, Isc.196, Isc.180 and Isc.179) identified in our study may be candidate target genes for tick control.

## Methods

### Sequence retrieval and characteristics identification

The search strategy used here is described as follow (Additional File 1). First, BPTI (a typical member of the Kunitz/BPTI family, UniProtKB AC: P00974) was used as a query sequence to search for Kunitz/BPTI protein sequences from all ticks against the NR database using BLASTP at the NCBI website under the default parameters [72,73]. Second, all hit sequences were filtered for the existence of a Kunitz/BPTI domain, which was determined by searching against the Pfam database under the default parameters [74]. To confirm that Kunitz/BPTI proteins were completely excavated, these putative Kunitz/BPTI proteins identified in this round of BLASTP search were used as a query to perform new rounds of BLASTP until no new hits appeared. Finally, a total of three rounds of BLASTP searches were performed, and there were 291, 368, and 368 sequences retrieved in the first, second and third rounds of the BLASTP search, respectively. Additionally, we used PSI-BLAST to search for Kunitz/BPTI proteins. These two search strategies have similar performance (Additional File 16). All sequences identified as Kunitz/BPTI proteins were submitted to the SignalP 4.0 server [30] for signal peptide prediction. Gene structures of the Kunitz/BPTI family were determined using the gene structure display server http://gsds.cbi.pku.edu.cn/index.php[75].

### Phylogenetic analysis of the Kunitz/BPTI family

To facilitate analysis of the Kunitz/BPTI family in ticks, Kunitz/BPTI proteins were classified into the following categories based their domain architectures: single-domain Kunitz/BPTI proteins present only in *Ixodes Scapularis *(ixosc_1domain), single-domain Kunitz/BPTI proteins present in all ticks (all_1domain), two-domain Kunitz/BPTI proteins present in all ticks (all_2domain), three-domain Kunitz/BPTI proteins present in all ticks (all_3domain), four-domain Kunitz/BPTI proteins present in all ticks (all_4domain), five-domain Kunitz/BPTI proteins present in all ticks (all_5domain), seven-domain Kunitz/BPTI proteins present in all ticks (all_7domain), and the combination of single-domain and multi-domain Kunitz/BPTI proteins present only in *Ixodes scapulari*s (ixosc_mix). Proteins in each category were aligned using ClustalW 2.0 [76] with fine adjustment by hand with reference to the cysteine residue position. Because the Kunitz domains are subjected to indels, ClustalW may not have treated these indels correctly. Therefore, the program Prank [77,78] was used to infer the indels and confirm the alignments obtained from ClustalW. Based on the alignment above, amino acid substitution models of protein evolution were determined by ProtTest 2.4 [79]. Phylogeny inference was performed using the neighbor-joining (NJ), Bayesian inference (MB) and maximum likelihood (ML) methods. MEGA 4.0 [80], MrBayes 3.1.2 [81] and Tree Finder [82] were used for reconstructing the NJ, MB and ML trees, respectively. All sets of parameters used for sequence alignment, model testing and phylogenetic analysis were listed (Additional File 17).

### Evolutionary analysis of the Kunitz/BPTI family

Three groups (groups I, II and III) from single-domain Kunitz/BPTI proteins in *Ixodes scapularis *were tested to determine whether they were subjected to selection. Tests of selection (nonsynonymous-to-synonymous rate ratio, ω = dN/dS) were accomplished by using codon-based substitution models implemented in the CODEML program of PAML version 4.4c [55]. Codon alignments of nucleotide sequences (Additional File 13) for three groups were constructed from the PAL2NAL server [83] based corresponding protein sequence alignments. The tree topology used for the three groups was based on the NJ trees in Additional File 14. The presence of a positively selected rate class was detected using the likelihood ratio tests (LRTs, i.e., by comparing the likelihood of a neutral model with that of a selection model). For detailed assumptions and descriptions of each model see papers [55,84]. The Model 0 (M0) assumes one ω for all sites. Three pairs of models make three LRTs by comparing M0 (one ratio) against M3 (discrete), M1a (nearly neutral model) against M2a (positive selection model) and M7 (βdistribution model) against M8 (β& ω model). To test for significance, the LRT statistic 2ΔL (twice the log likelihood difference) was compared against the *chi*-square distribution (with df = 2 with critical values of 9.21 for M1a vs. M2a and M7 vs. M8, but with df = 4 with critical values 13.28 for M0 vs. M3) at the 1% significance level. The Bayes Empirical Bayes (BEB) approach was used to calculate the posterior probability that each site was from a particular site class, and sites with high posterior probabilities coming from the class with ω > 1 (with p > 0.95) were inferred to be under positive selection. All analyses were run twice using different initial ω values to ensure convergence. Substitution saturation could bias the estimation of the dN/dS ratios. In order to assess substitution saturation of three groups (group I, II and III), two methods (Steel's method [56] and Xia's method [57]) implemented in the software DAMBE [58] were used.

### Protein modeling, structural alignment and locating positively selected sites

Nine proteins (XP_002406145 from group I; AAM93606, AAM93607, AAM93608, AAM93610 and AAM93612 from group II; AAM93632, AAM93635 and AAK97828 from group III) were modeled using the I-TASSER server [62,85]. All model structures predicted here had been deposited in Protein Model DataBase [86] under ID numbers PM0077184-PM0077186 and PM0077307-PM0077312. Structural alignment was performed using the MISTRAL online server [87]. PyMOL [88] was used to visualize the structure and locate the positively selected sites.

### Comparison of the expression levels in different stages of blood feeding

To estimate the differential expression of *Ixodes scapularis *genes in the different stages of blood feeding, four cDNA libraries from *Ixodes scapularis *adult female ticks, which were constructed following the same protocol described in a previous paper [15], were downloaded from the NCBI website for *in silico *analysis. The four cDNA libraries are as follows: 1) adult females, unfed (ISUF); 2) adult females, 6-12 hours after host attachment (IS6-12); 3) adult females, 18-24 hours after host attachment (IS18-24); and 4) adult females, 3-4 days after host attachment (ISA72). Details of the libraries were shown (Additional File 18). Then these four cDNA libraries were analyzed using R statistics [53], which is a method based on the comparison of the abundance of ESTs contributing to each contig in the different cDNA libraries. This method allows for the comparison of gene expression in any number of libraries, and it can identify differentially expressed genes as those with a high R-value. A Perl program was written to conduct this analysis (Additional File 13). The expression of *Ixodes ricinus *genes in the different stages of blood feeding was also analyzed. Because the cDNA libraries and Unigene database for *Ixodes ricinus *are unavailable, R statistics is not applicable in this case. We searched for Kunitz/BPTI sequences against the EST database of *Ixodes ricinus *in NCBI by TBLASTN. Based the annotation of the ESTs, we obtained gene expression information for the Kunitz/BPTI family in the different stages of blood feeding.

## Authors' contributions

SXD and ADZ carried out the data mining, the sequence alignments, bioinformatic analysis and wrote the manuscript. JFH designed and coordinated the study and participated in writing the manuscript. All authors read and approved the final manuscript.

## Supplementary Material

Additional file 1 **Figure S1**. Two strategies for database searches of ticks Kunitz/BPTI proteins from NR database in NCBI. Click here for file

Additional file 2 **Table S1**. Details of removed protein sequences. Click here for file

Additional file 3 **Table S2**. Detailed information for all 303 sequences. Click here for file

Additional file 4 **Figure S2**. Neighbor-joining (NJ) and maximum likelihood (ML) tree of single-domain Kunitz/BPTI proteins in *Ixodes scapularis*. Click here for file

Additional file 5 **Figure S3**. Alignment of single-domain Kunitz/BPTI proteins in *Ixodes scapularis*. Click here for file

Additional file 6 **Figure S4**. Neighbor-joining (NJ) and maximum likelihood (ML) tree of all single-domain Kunitz/BPTI proteins in ticks. Click here for file

Additional file 7 **Figure S5**. Alignment of group II and group III Kunitz/BPTI protein sequences translated from ESTs in the *Ixodes ricinus*. Click here for file

Additional file 8 **Figure S6**. Bayesian inference (MB) tree of single and multi-domain Kunitz/BPTI proteins in *Ixodes scapularis*. Click here for file

Additional file 9 **Table S3**. Results of selection test for group II. Click here for file

Additional file 10 **Table S4**. Results of selection test for group III. Click here for file

Additional file 11 **Table S5**. Results of selection test for group I. Click here for file

Additional file 12 **S1**. Results for assessing substitution saturation of the three groups. Click here for file

Additional file 13 **S2**. Codon alignments of nucleotide sequences for three groups and script of the Perl program used in our study. Click here for file

Additional file 14 **Figure S7**. Phylogeny of group I, II and III and for PAML analyze with dN and dS on each branch. Click here for file

Additional file 15 **Figure S8**. Alignment of Ra-KLP related proteins in Metastriata ticks and group II proteins in Prostriata ticks. Click here for file

Additional file 16 **Table S6**. Two search strategies have similar performance. Click here for file

Additional file 17 **Table S7**. All sets of parameters used for sequence and phylogenetic analysis. Click here for file

Additional file 18 **Table S8**. Details of the four cDNA libraries used for the expression analysis. Click here for file
