# Supplementary material for: Evolution, expansion and expression of the Kunitz/BPTI gene family associated with long-term blood feeding in Ixodes Scapularis
Source: BMC Evol Biol. 2012 Jan 14;12:4. doi: 10.1186/1471-2148-12-4 (PMC3273431; doi:10.1186/1471-2148-12-4)
Supplement: Additional file 1 — Figure S1. Two strategies for database searches of ticks Kunitz/BPTI proteins from NR database in NCBI. [file 1471-2148-12-4-S1.DOC]

BPTI (UniProtKB AC P00974) BPTI (UniProtKB AC P00974)

[**PSI - BLAST**](http://www.ncbi.nlm.nih.gov/blast/Blast.cgi?CMD=Web&LAYOUT=TwoWindows&AUTO_FORMAT=Semiauto&ALIGNMENTS=250&ALIGNMENT_VIEW=Pairwise&CLIENT=web&COMPOSITION_BASED_STATISTICS=on&DATABASE=nr&CDD_SEARCH=on&DESCRIPTIONS=500&ENTREZ_QUERY=(none)&EXPECT=10&FORMAT_OBJECT=Alignment&FORMAT_TYPE=HTML&I_THRESH=0.005&MATRIX_NAME=BLOSUM62&NCBI_GI=on&PAGE=Proteins&PROGRAM=blastp&RUN_PSIBLAST=on&SERVICE=plain&SET_DEFAULTS.x=36&SET_DEFAULTS.y=5&SHOW_OVERVIEW=on&END_OF_HTTPGET=Yes&SHOW_LINKOUT=yes&GET_SEQUENCE=yes)

[**BLAST**](http://www.ncbi.nlm.nih.gov/blast/Blast.cgi?CMD=Web&LAYOUT=TwoWindows&AUTO_FORMAT=Semiauto&ALIGNMENTS=250&ALIGNMENT_VIEW=Pairwise&CLIENT=web&COMPOSITION_BASED_STATISTICS=on&DATABASE=nr&CDD_SEARCH=on&DESCRIPTIONS=500&ENTREZ_QUERY=(none)&EXPECT=10&FORMAT_OBJECT=Alignment&FORMAT_TYPE=HTML&I_THRESH=0.005&MATRIX_NAME=BLOSUM62&NCBI_GI=on&PAGE=Proteins&PROGRAM=blastp&RUN_PSIBLAST=on&SERVICE=plain&SET_DEFAULTS.x=36&SET_DEFAULTS.y=5&SHOW_OVERVIEW=on&END_OF_HTTPGET=Yes&SHOW_LINKOUT=yes&GET_SEQUENCE=yes)**P**

**Protein sequences of *ticks***

**Protein sequences of *ticks***

NR database in NCBI NR database in NCBI

**5 iteration**

All hit sequences

All hit sequences

filtered by the existence of Kunitz/BPTI domain

filtered by the existence of Kunitz/BPTI domain

**PFAM**

**PFAM**

All Kunitz/BPTI proteins

364 Kunitz/BPTI proteins

**3 rounds of BLASTP no new hits appeared**

368 Kunitz/BPTI proteins

## Figure S1. Two strategies for database searches of ticks Kunitz/BPTI proteins from NR database in NCBI

Two strategies for database searches of ticks Kunitz/BPTI proteins from NR database in NCBI were shown. A total of three rounds of BLASTP search and 6 iterations of PSI-BLAST were performed.
