# Supplementary material for: Evolution, expansion and expression of the Kunitz/BPTI gene family associated with long-term blood feeding in Ixodes Scapularis
Source: BMC Evol Biol. 2012 Jan 14;12:4. doi: 10.1186/1471-2148-12-4 (PMC3273431; doi:10.1186/1471-2148-12-4)
Supplement: Additional file 2 — Table S1. Details of removed protein sequences. [file 1471-2148-12-4-S2.DOC]

**Table S1. Details of removed protein sequences**

| Removed sequence | Retained sequence | Percentage identity | Unigene |
| --- | --- | --- | --- |
| AAM93611 | AAY66615 | 91.79 | Isc.218 |
| AAY66732 | AAM93613 | 97.89 | Isc.188 |
| AAY66715 | AAY66764 | 92.31 | Isc.136 |
| AAY66727 | AAM93630 | 96.7 | Isc.158 |
| AAM93615 | AAY66724 | 97.87 | Isc.230 |
| AAM93603 | AAY66724 | 92 | Isc.230 |
| AAY66710 | AAM93610 | 94.05 | Isc.196 |
| AAY66702 | AAM93635 | 90 | Isc.180 |
| AAM93634 | AAM93635 | 98.96 | Isc.180 |
| AAM93631 | AAM93632 | 98.96 | Isc.179 |
| AAM93605 | AAM93606 | 97.56 | Isc.190 |

Note. To identify Kunitz/BPTI proteins in *Ixodes scapularis*, we carried out BLASTP and TBLASTN iteratively until no new hits appeared (see Materials and Methods). This powerful method identified 91 sequences. Some pairs of these sequences are clustered in the same Unigene and show more than 90% identity. Each pairs of sequences may be coded by the same gene, as they cluster in the same Unigene. Therefore, in each pair, we retained only one sequence which has more ESTs support.
