# Supplementary material for: Evolution, expansion and expression of the Kunitz/BPTI gene family associated with long-term blood feeding in Ixodes Scapularis
Source: BMC Evol Biol. 2012 Jan 14;12:4. doi: 10.1186/1471-2148-12-4 (PMC3273431; doi:10.1186/1471-2148-12-4)
Supplement: Additional file 4 — Figure S2. Neighbor-joining (NJ) and maximum likelihood (ML) tree of single-domain Kunitz/BPTI proteins in Ixodes scapularis. [file 1471-2148-12-4-S4.DOC]

##
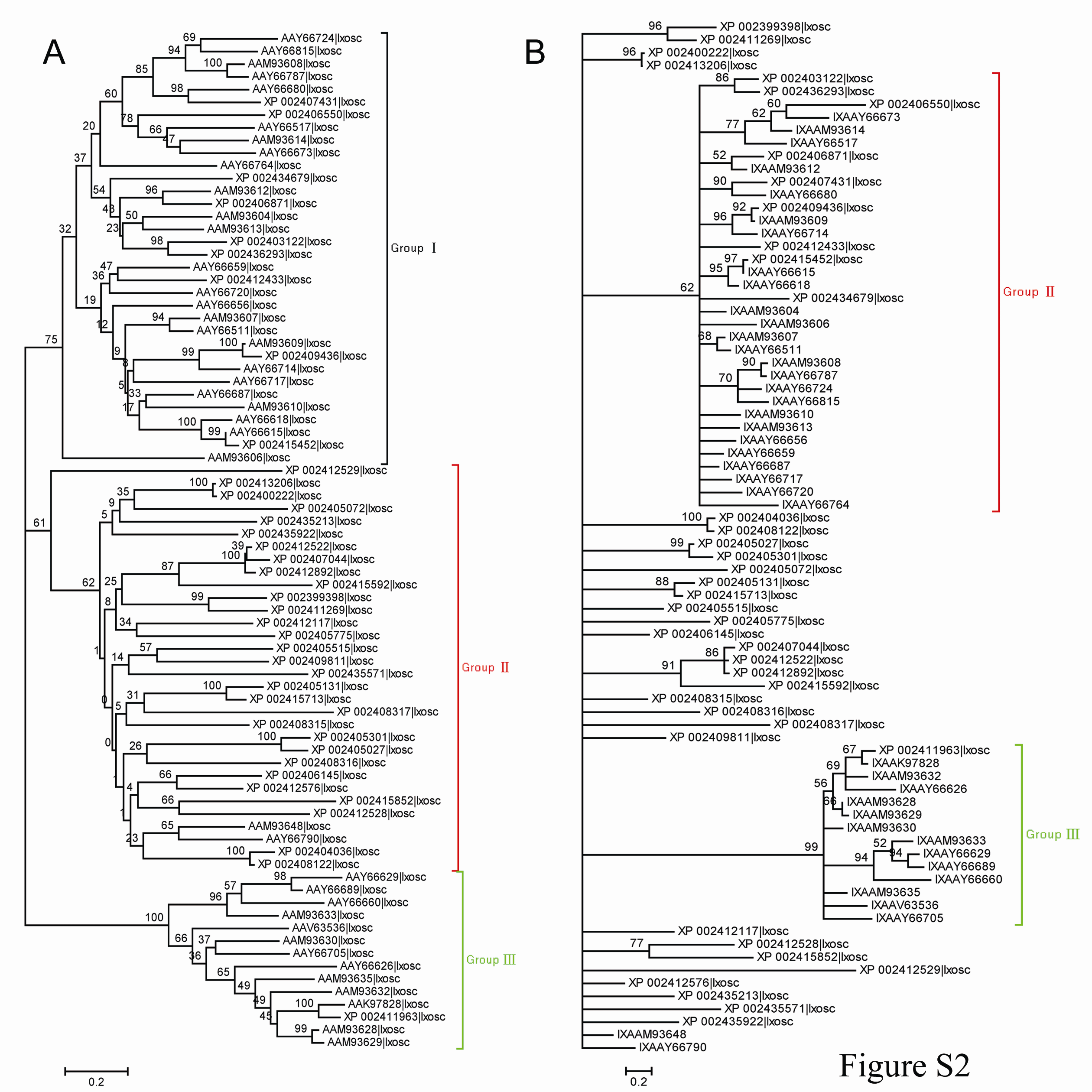


## Figure S2. Neighbor-joining (NJ) and maximum likelihood (ML) tree of single-domain Kunitz/BPTI proteins in *Ixodes scapularis*

Neighbor-joining (NJ) tree **(A)** and maximum likelihood (ML) tree **(B)** for single-domain Kunitz/BPTI proteins in *Ix**odes scapularis* were shown. The length of each branch is proportional to the estimated number of substitutions. Bar denotes 20% substitutions per site. The Kunitz/BPTI family is divided into three groups (in NJ tree, but not in MB tree), which are colored as follows: black, group I; red, group II; green, group III and marked with circle, square and triangle, respectively. Three groups show different cysteine patterns: CX(8)CX(15)CX(7)CX(12)CX(3)C for group I, CX(8)CX(18)CX(5)CX(12)CX(3)C for group II, CX(5,6)CX(15)CX(8)CX(11)CX(3)C for group III, respectively.
