# Supplementary material for: Evolution, expansion and expression of the Kunitz/BPTI gene family associated with long-term blood feeding in Ixodes Scapularis
Source: BMC Evol Biol. 2012 Jan 14;12:4. doi: 10.1186/1471-2148-12-4 (PMC3273431; doi:10.1186/1471-2148-12-4)
Supplement: Additional file 5 — Figure S3. Alignment of single-domain Kunitz/BPTI proteins in Ixodes scapularis. [file 1471-2148-12-4-S5.DOC]

##
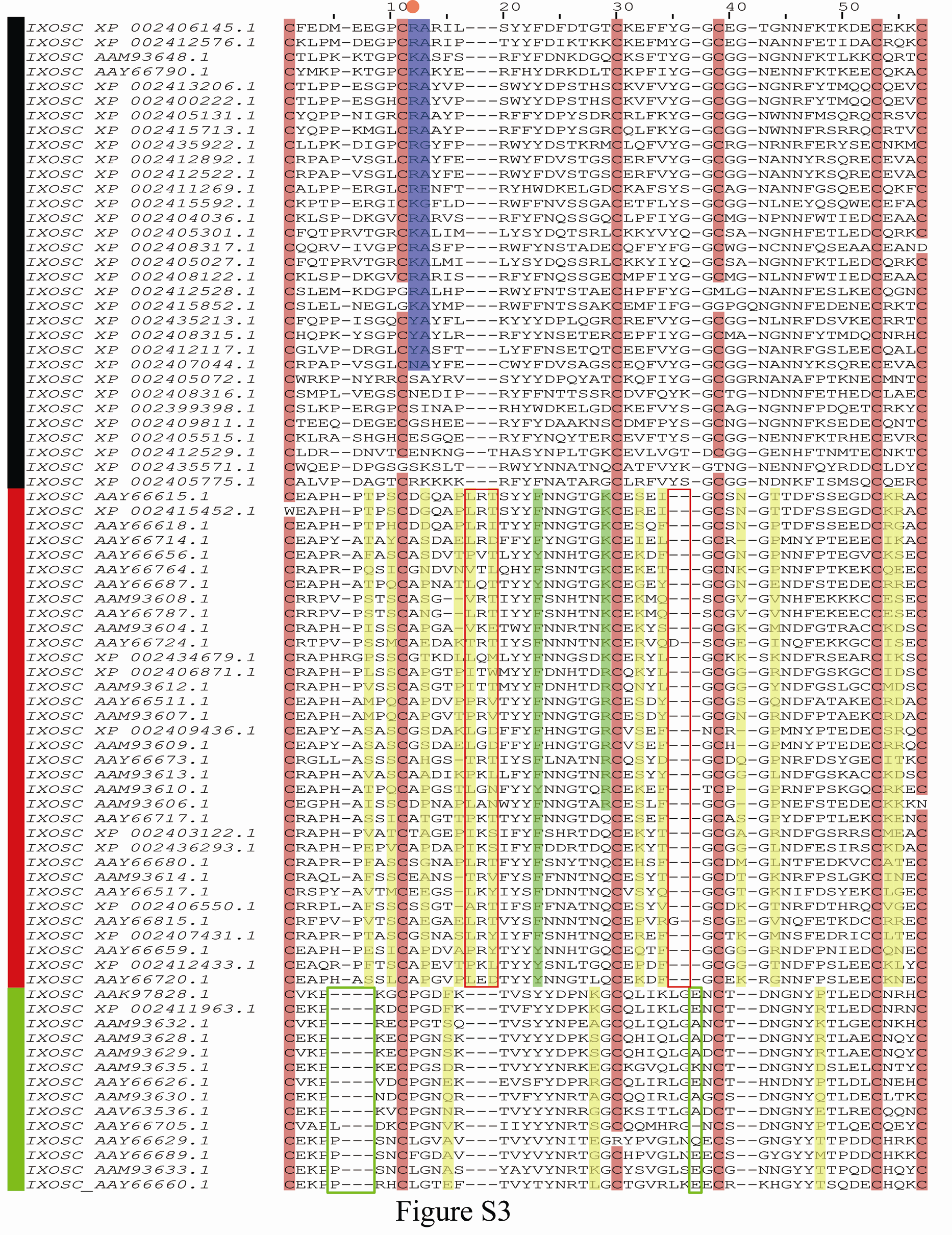


## Figure S3 Alignment of single-domain Kunitz/BPTI proteins in *Ixodes scapularis*

The alignment only shows the part between the first and sixth cysteine (C1-C6) in Kunitz/BPTI protein. Three groups are indicated by colored lines at left: black, group I; red, group II; green, group III. The six conserved cysteines are colored by red. Colored boxes indicate different indels relative to group I: red, group II type indels; green, group III type indels. Red circle indicates the position of P1 site. Key residues for serine proteases inhibition and ion channels modulating are highlighted in blue and green, respectively. Positively selected sites in group II and III are colored by yellow.
