# Supplementary material for: Evolution, expansion and expression of the Kunitz/BPTI gene family associated with long-term blood feeding in Ixodes Scapularis
Source: BMC Evol Biol. 2012 Jan 14;12:4. doi: 10.1186/1471-2148-12-4 (PMC3273431; doi:10.1186/1471-2148-12-4)
Supplement: Additional file 6 — Figure S4. Neighbor-joining (NJ) and maximum likelihood (ML) tree of all single-domain Kunitz/BPTI proteins in ticks. [file 1471-2148-12-4-S6.DOC]

##
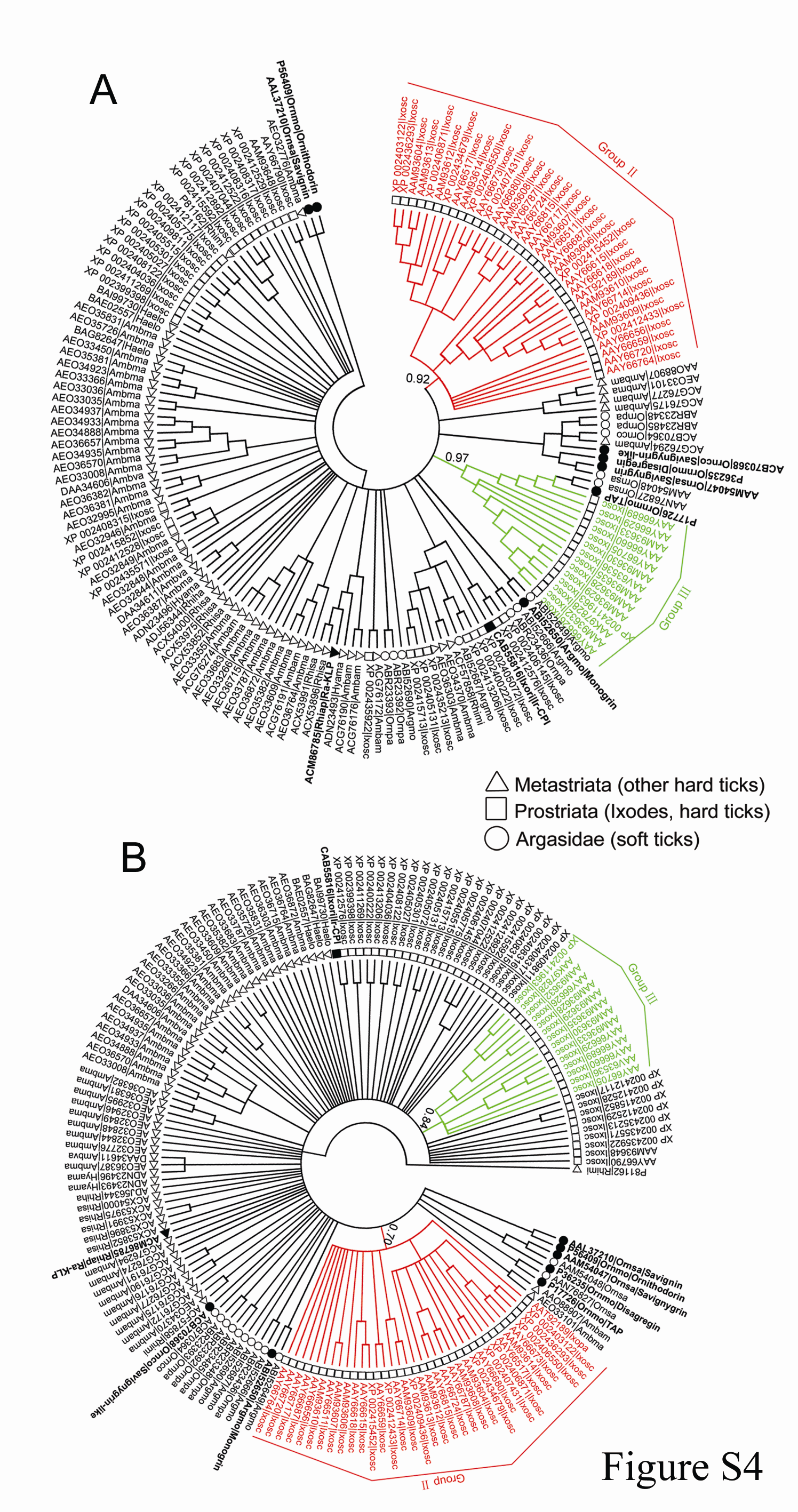


## Figure S4. Neighbor-joining (NJ) and maximum likelihood (ML) tree of all single-domain Kunitz/BPTI proteins in ticks

The circular phylogram of ML tree **(A)** and NJ tree **(B)** are based on alignment of Kunitz/BPTI proteins in *Ixodes scapularis* and other ticks. Group II and group III, which are highlighted, are lineage-specific expansions of Kunitz/BPTI family in the genus *Ixodes*. The sequence name consists of 5 letters (3 from the genus and 2 from the specie name) followed by the NCBI accession number. Circle, square and triangle in the phylogram indicate the proteins from soft ticks, the genus *Ixodes* (hard ticks) and other hard ticks, respectively. (Argmo, *Argas monolakensis*; Ornco, *Ornithodoros coriaceus*; Ornmo, *Ornithodoros moubata*; Ornpa, *Ornithodoros parkeri*; Ornsa, *Ornithodoros savignyi*; Rhiap, *Rhipicephalus appendiculatus*; Rhisa, *Rhipicephalus sanguineus*; Ixosc, *Ixodes scapularis*; Ixopa, *Ixodes pacificus*; Ixori, *Ixodes ricinus*; Rhimi, *Rhipicephalus microplus*; Hyama, *Hyalomma marginatum rufipes*; Ambam, *Amblyomma americanum*; Haelo, *Haemaphysalis longicornis*)
