# Supplementary material for: Evolution, expansion and expression of the Kunitz/BPTI gene family associated with long-term blood feeding in Ixodes Scapularis
Source: BMC Evol Biol. 2012 Jan 14;12:4. doi: 10.1186/1471-2148-12-4 (PMC3273431; doi:10.1186/1471-2148-12-4)
Supplement: Additional file 7 — Figure S5. Alignment of group II and group III Kunitz/BPTI protein sequences translated from ESTs in the Ixodes ricinus. [file 1471-2148-12-4-S7.DOC]

##
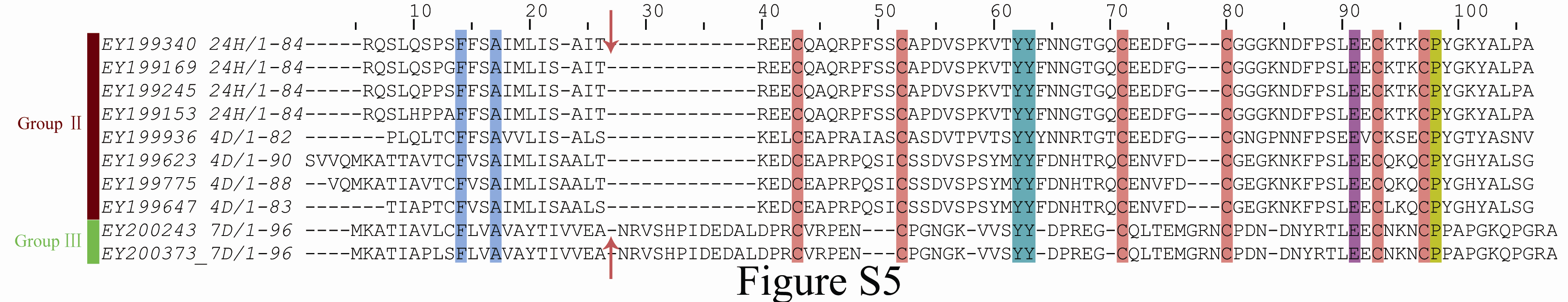


## Figure S5. Phylogeny of group I for PAML analyze with dN and dS on each branch

The phylogenetic tree of 32 sequences in group I was used for PAML analysis. The values of dN and dS were labeled in the branches.
