# Supplementary material for: Evolution, expansion and expression of the Kunitz/BPTI gene family associated with long-term blood feeding in Ixodes Scapularis
Source: BMC Evol Biol. 2012 Jan 14;12:4. doi: 10.1186/1471-2148-12-4 (PMC3273431; doi:10.1186/1471-2148-12-4)
Supplement: Additional file 8 — Figure S6. Bayesian inference (MB) tree of single and multi-domain Kunitz/BPTI proteins in Ixodes scapularis. [file 1471-2148-12-4-S8.DOC]

##
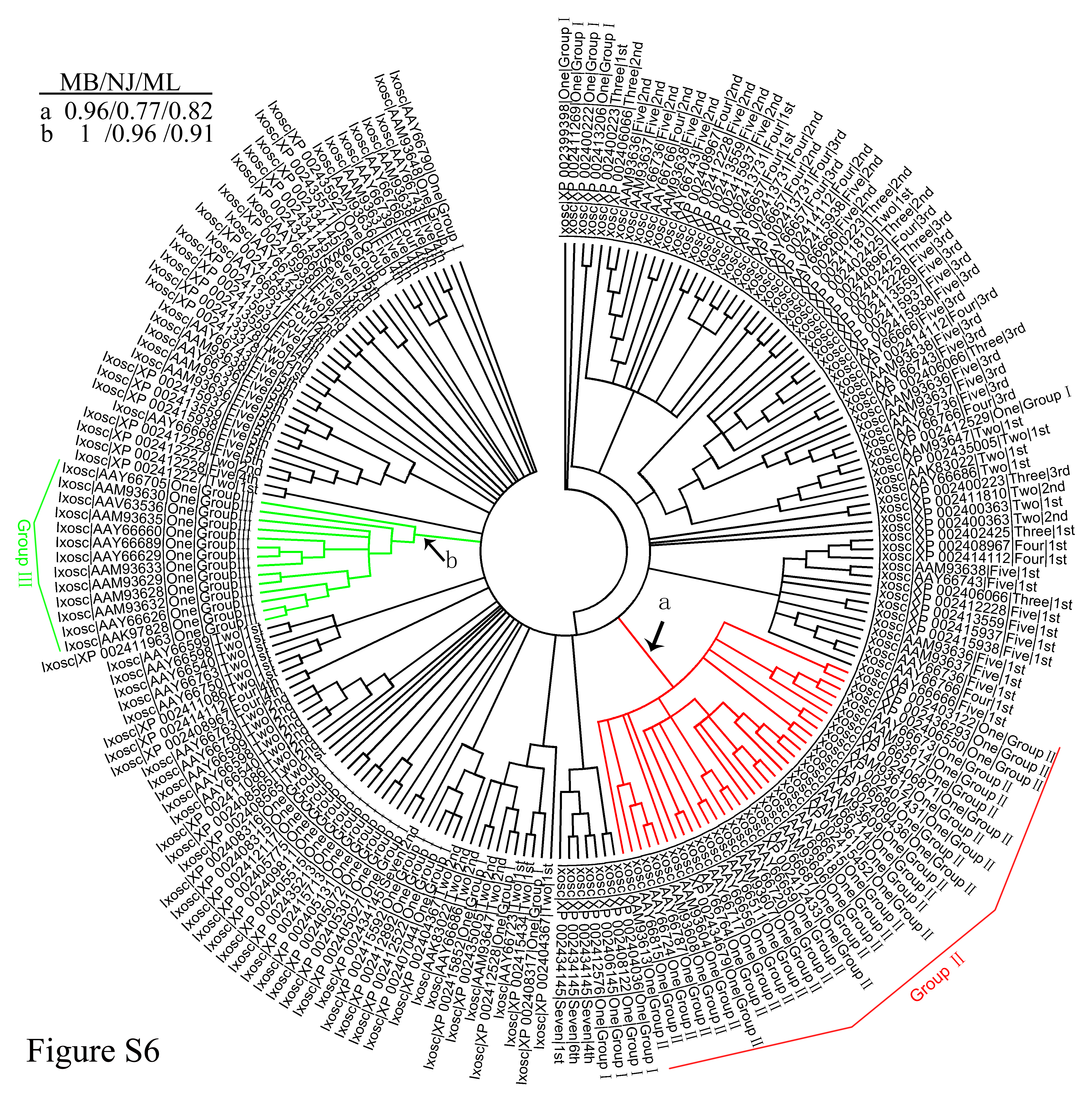


## Figure S6. Bayesian inference (MB) tree of single and multi-domain Kunitz/BPTI proteins in *Ixodes scapularis*

All multi-domain Kunitz/BPTI proteins in *Ixodes scapularis* were split into single-domain segments. Then these single-domain segments were aligned together with all single-domain Kunitz/BPTI proteins in *Ixodes scapularis* and their corresponding phylogenetic trees were constructed. Group II and group III, which are highlighted, form two separate monophyletic clades, respectively, with highly support. The support values for the two groups (in MB, NJ, and ML tree) were shown in the left top of figure.
