# Supplementary material for: Evolution, expansion and expression of the Kunitz/BPTI gene family associated with long-term blood feeding in Ixodes Scapularis
Source: BMC Evol Biol. 2012 Jan 14;12:4. doi: 10.1186/1471-2148-12-4 (PMC3273431; doi:10.1186/1471-2148-12-4)
Supplement: Additional file 9 — Table S3. Results of selection test for group II. [file 1471-2148-12-4-S9.DOC]

**Table S3. Results of selection test for group II**

| **Model** | **L** | **Estimates of parameters** | **2△L** | **P-value** | **Positively selected sites** |
| --- | --- | --- | --- | --- | --- |
| **M0**  **(one ratio)** | -5184.245496（67） | ω= 0.76433 |  |  | None |
| **M3 (discrete)** | -4934.937415（71） | P_0_=0.22252,ω_0_=0.11252  P_1_=0.38573,ω_1_=0.82956  P_2_=0.39175, ω_2_=2.39442 | 498.62 | <0.0001 | * |
| **M1 (neutral)** | -4986.444772 (68) | P_0_=0.26682,ω_0_= 0.73318  P_1_= 0.12079,ω_1_=1.00 |  |  | Not allowed |
| **M2 (selection)** | -4936.638771 (70) | P_0_=0.22946,ω_0_=0.13085  P_1_= 0.39548, ω_1_=1.00  P_2_=0.37505,ω_2_=2.71355 | 99.61 | <0.0001 | 21S 32M 34Q 37P 40T 41P 43V 44T 56S 58Y 63N 65R 75R 76D 88S |
| **M7 (β)** | -4975.018028 (68) | P= 0.41883, q= 0.21638 |  |  | Not allowed |
| **M8 (β& ω)** | -4934.171877 (70) | P_1_=0.37763,ω= 2.36730  P_0_= 0.62237  P= 0.51811, q= 0.38018 | 81.69 | <0.0001 | 21S 32M 34Q 37P 40T 41P 43V 56S 58Y 63N 65R 75R 76D 88S |

Note: Numbers in parentheses represent the number of parameters in the ω distribution. 2△L and P-value are for comparison three pairs: M0/M3, M1/M2 and M7/M8. Positively selected sites with posterior probabilities (P) > 0.95 under Bayes Empirical Bayes (BEB) analysis are shown in this table. The amino acids refer to AAM93607.1.

* Bayes Empirical Bayes (BEB) analysis is not implemented based M3 (discrete), so positively selected sites detected by the model are not shown in this table.
