# Supplementary material for: Evolution, expansion and expression of the Kunitz/BPTI gene family associated with long-term blood feeding in Ixodes Scapularis
Source: BMC Evol Biol. 2012 Jan 14;12:4. doi: 10.1186/1471-2148-12-4 (PMC3273431; doi:10.1186/1471-2148-12-4)
Supplement: Additional file 10 — Table S4. Results of selection test for group III. [file 1471-2148-12-4-S10.DOC]

**Table S4. Results of selection test for group III**

| **Model** | **L** | **Estimates of parameters** | **2△L** | **P-value** | **Positively selected sites** |
| --- | --- | --- | --- | --- | --- |
| **M0**  **(one ratio)** | -2188.964317（27） | ω= 0.73799 |  |  | None |
| **M3 (discrete)** | -2128.592256（31） | P_0_=0.33348,ω_0_=0.14115  P_1_=0.57760,ω_1_=1.07752  P_2_=0.08892,ω_2_=4.30578 | 120.74 | <0.0001 | * |
| **M1 (neutral)** | -2140.842190 (28) | P_0_=0.38011,ω_0_=0.14212  P_1_= 0.61989, ω_1_=1.00 |  |  | Not allowed |
| **M2 (selection)** | -2128.678292 (30) | P_0_=0.31909,ω_0_=0.12689  P_1_= 0.58208, ω_1_=1.00  P_2_=0.09883,ω_2_=3.88713 | 24.33 | <0.0001 | 31F 41K 59P |
| **M7 (β)** | -2141.049423 (28) | P= 0.39319, q= 0.23670 |  |  | Not allowed |
| **M8 (β& ω)** | -2128.811385 (30) | P_1_=0.11249,ω= 3.35169  P_0_= 0.88751  P= 0.38713, q= 0.22988 | 24.48 | <0.0001 | 31F 41K 59P |

Note: Numbers in parentheses represent the number of parameters in the ω distribution. 2△L and P-value are for comparison three pairs: M0/M3, M1/M2 and M7/M8. Positively selected sites with posterior probabilities (P) > 0.95 under Bayes Empirical Bayes (BEB) analysis are shown in this table. The amino acids refer to AAK97828.1.

* Bayes Empirical Bayes (BEB) analysis is not implemented based M3 (discrete), so positively selected sites detected by the model are not shown in this table.
