# Supplementary material for: Evolution, expansion and expression of the Kunitz/BPTI gene family associated with long-term blood feeding in Ixodes Scapularis
Source: BMC Evol Biol. 2012 Jan 14;12:4. doi: 10.1186/1471-2148-12-4 (PMC3273431; doi:10.1186/1471-2148-12-4)
Supplement: Additional file 11 — Table S5. Results of selection test for group I. [file 1471-2148-12-4-S11.DOC]

**Table S5. Results of selection test for group** I

| **Model** | **L** | **Estimates of parameters** | **2△L** | **P-value** | **Positively selected sites** |
| --- | --- | --- | --- | --- | --- |
| **M0**  **(one ratio)** | -4462.261425（63） | ω= 0.16223 |  |  | None |
| **M3 (discrete)** | -4164.587904（67） | P_0_=0.25773,ω_0_=0.01163  P_1_=0.15168,ω_1_=0.08337  P_2_=0.59058,ω_2_=0.37778 | 595.35 | <0.0001 | None |
| **M1 (neutral)** | -4230.094509 (64) | P_0_=0.35745,ω_0_=0.05340  P_1_= 0.64255, ω_1_=1.000 |  |  | Not allowed |
| **M2 (selection)** | -4230.094509 (66) | P_0_=0.35745,ω_0_=0.05340  P_1_= 0.55598, ω_1_=1.00  P_2_= 0.08657, ω_2_=1.00 | 0 | 1 | None |
| **M7 (β)** | -4165.488992 (64) | P=0.57213, q=1.60139 |  |  | Not allowed |
| **M8 (β& ω)** | -4165.489208 (66) | P_1_= 0.00001, ω=1.00  P_0_=0.92  P=0.57214, q=1.60140 | 0 | 1 | None |

Note: Numbers in parentheses represent the number of parameters in the ω distribution. 2△L and P-value are for comparison three pairs: M0/M3, M1/M2 and M7/M8. Positively selected sites with posterior probabilities (P) > 0.95 under Bayes Empirical Bayes (BEB) analysis are shown in this table. The amino acids refer to XP_002406145.1.
