# Supplementary material for: Evolution, expansion and expression of the Kunitz/BPTI gene family associated with long-term blood feeding in Ixodes Scapularis
Source: BMC Evol Biol. 2012 Jan 14;12:4. doi: 10.1186/1471-2148-12-4 (PMC3273431; doi:10.1186/1471-2148-12-4)
Supplement: Additional file 14 — Figure S7. Phylogeny of group I, II and III and for PAML analyze with dN and dS on each branch. [file 1471-2148-12-4-S14.DOC]

##
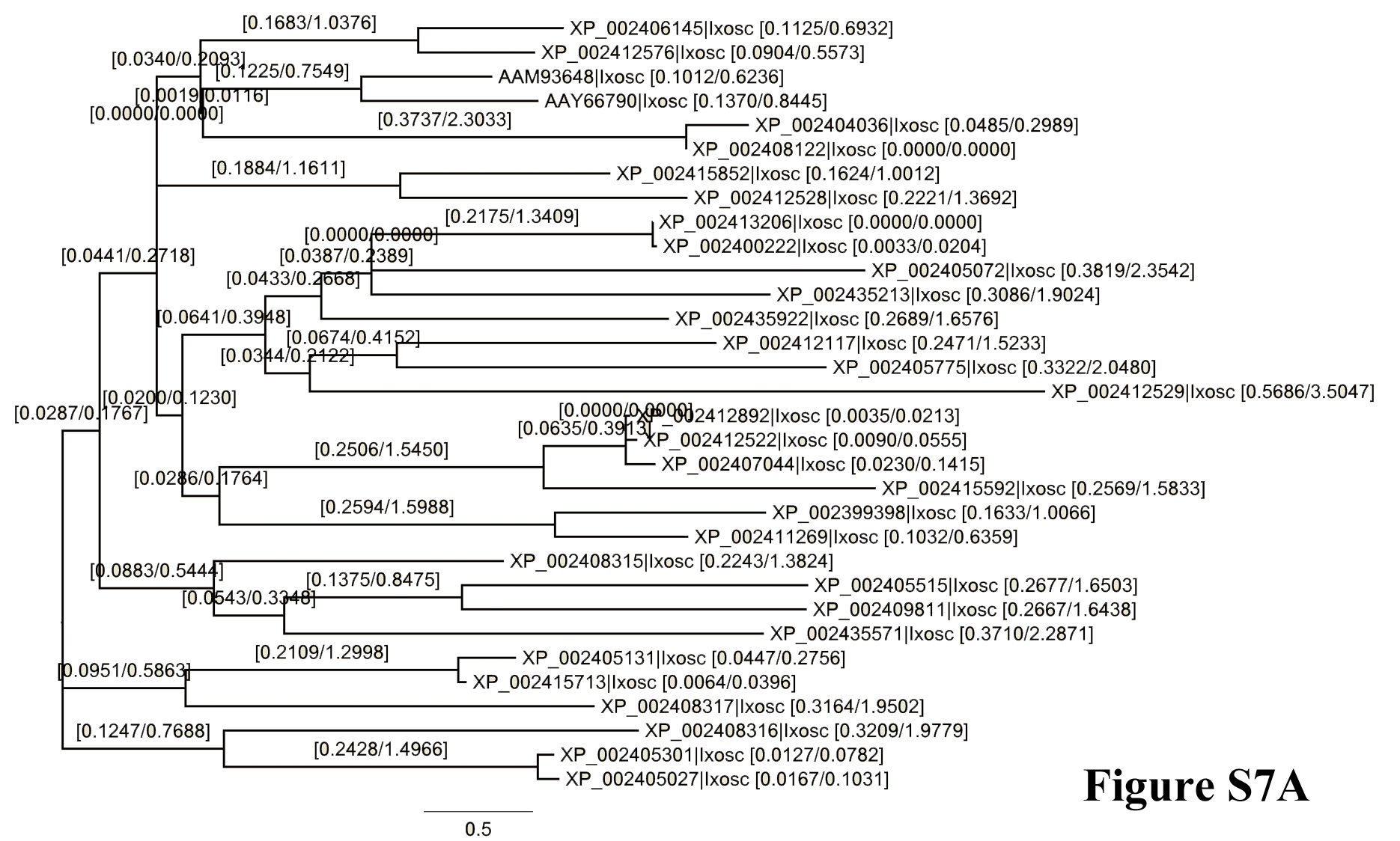

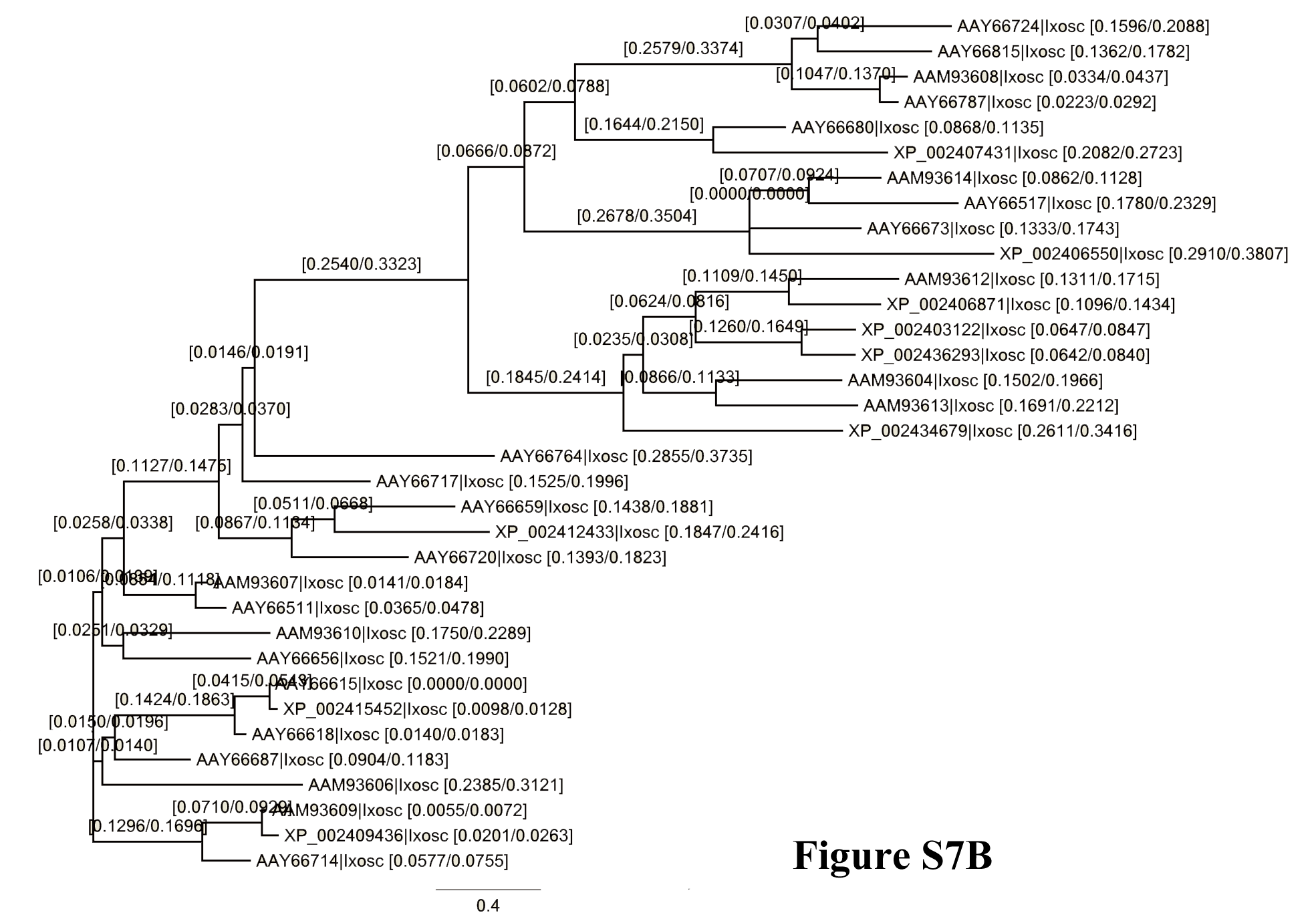


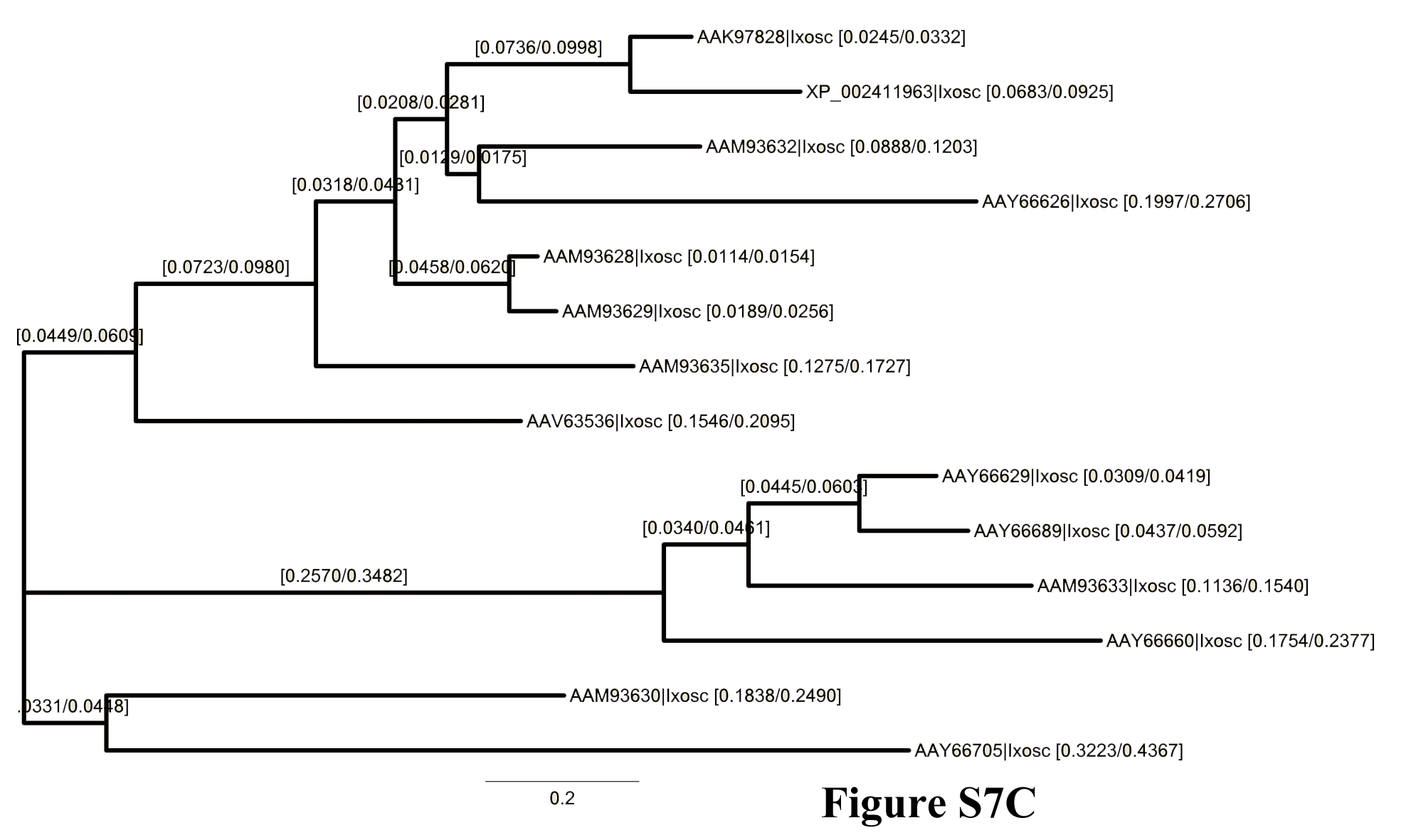


## Figure S7. Phylogeny of group I, II and III for PAML analyze with dN and dS on each branch

The phylogenetic trees of group I (Figure S7A), group II (Figure S7B) and group III (Figure S7C) were used for PAML analysis. The values of dN and dS were labeled in the branches.
