# Supplementary material for: Evolution, expansion and expression of the Kunitz/BPTI gene family associated with long-term blood feeding in Ixodes Scapularis
Source: BMC Evol Biol. 2012 Jan 14;12:4. doi: 10.1186/1471-2148-12-4 (PMC3273431; doi:10.1186/1471-2148-12-4)
Supplement: Additional file 15 — Figure S8. Alignment of Ra-KLP related proteins in Metastriata ticks and group II proteins in Prostriata ticks. [file 1471-2148-12-4-S15.DOC]

##
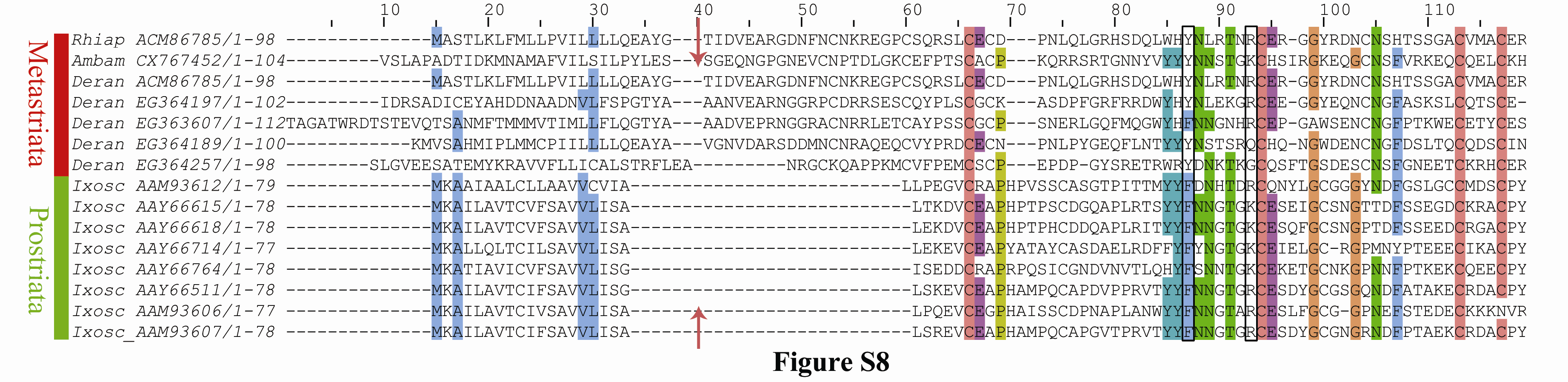


## Figure S8. Alignment of Ra-KLP related proteins in Metastriata ticks and group II proteins in Prostriata ticks

Different origins of proteins are indicated by colored lines at left: red, Metastriata ticks; green, Prostriata ticks. The arrows indicate the signal peptide cleavage sites. Residues in black boxes are associated with channel-modulating activity. (Rhiap, *Rhipicephalus appendiculatus*; Ambam, *Amblyomma americanum*; Deran, *Dermacentor andersoni*)
