# Supplementary material for: Evolution, expansion and expression of the Kunitz/BPTI gene family associated with long-term blood feeding in Ixodes Scapularis
Source: BMC Evol Biol. 2012 Jan 14;12:4. doi: 10.1186/1471-2148-12-4 (PMC3273431; doi:10.1186/1471-2148-12-4)
Supplement: Additional file 16 — Table S6. Two search strategies have similar performance. [file 1471-2148-12-4-S16.DOC]

**Table S6.** **Two search strategies have similar performance**

Note. Comparison of BLASTP and PSI-BLAST for database searches of ticks Kunitz/BPTI proteins from NR database in NCBI. A total of **three** rounds of BLASTP search and **6 iterations** of PSI-BLAST were performed. The number of retrieved sequences for each search was shown. Two search strategies have similar performance

|  | **1** | **2** | **3** |  | 5 | 6 |  |
| --- | --- | --- | --- | --- | --- | --- | --- |
| BLASTP | **291** | **368** | **368** |  |  |  |  |
| PSI-BLAST |  |  |  |  | 364 | 356 |  |
