# Supplementary material for: Evolution, expansion and expression of the Kunitz/BPTI gene family associated with long-term blood feeding in Ixodes Scapularis
Source: BMC Evol Biol. 2012 Jan 14;12:4. doi: 10.1186/1471-2148-12-4 (PMC3273431; doi:10.1186/1471-2148-12-4)
Supplement: Additional file 18 — Table S8. Details of the four cDNA libraries used for the expression analysis. [file 1471-2148-12-4-S18.DOC]

**Table S8. Details of the four cDNA libraries used for the expression analysis**

| Library ID | Library Name | Number of ESTs  sequenced | Number of  Unigene clusters |
| --- | --- | --- | --- |
| Lib.17364 | ISUF | 1026 | 490 |
| Lib.17366 | IS6-12 | 952 | 413 |
| Lib.17365 | IS18-24 | 1995 | 459 |
| Lib.17361 | ISA72 | 1993 | 744 |

Note. The four libraries used for expression analysis are all from NCBI website (http://www.ncbi.nlm.nih.gov/unigene/). They were constructed by use of the same protocol . Each library represents a different stage of blood-feeding in Ixodes scapularis.

1. Ribeiro, J.M., et al., *An annotated catalog of salivary gland transcripts from Ixodes scapularis ticks.* Insect Biochem Mol Biol, 2006. **36**(2): p. 111-29.
